# Resilience of *Portulaca* plants to environmental stresses and the economic potential of their bioactive compounds

**DOI:** 10.1007/s00425-025-04767-1

**Published:** 2025-07-15

**Authors:** Yong Chie Liew, Lucas Wei Tze Lim, Su-Ee Lau, Boon Chin Tan

**Affiliations:** 1 Faculty of Science, Universiti Malaya, 50603 Kuala Lumpur, Malaysia; 2 School of Biological Sciences, Universiti Sains Malaysia, 11800 George Town, Malaysia; 3 Centre for Research in Biotechnology for Agriculture, Universiti Malaya, 50603 Kuala Lumpur, Malaysia

**Keywords:** Omega-3 fatty acids, Flavonoids, Alkaloids, C_4_, Crassulacean acid metabolism (CAM) photosynthesis

## Abstract

**Main conclusion:**

This review highlights the health benefits and environmental potential of *Portulaca* species, particularly *Portulaca oleracea* L., and emphasizes addressing challenges in bioactive compound yields and scalability for broader applications.

**Abstract:**

*Portulaca* species (*Portulaca* spp.) are globally distributed and rich in bioactive compounds, including alkaloids, flavonoids, betalains, and fatty acids. These compounds exhibit antioxidant, antibacterial, anti-inflammatory, and anticancer activities. Among these species, *Portulaca oleracea* L. or purslane is notable for its long history in the traditional medicine. Its aqueous extracts have demonstrated anti-inflammatory, antidiabetic, and antioxidant properties, particularly in mitigating oxidative stress and gastrointestinal disorders. In addition, its nutritional profile, rich in omega-3 fatty acids, vitamins, and minerals, is higher than many leafy vegetables. As a genus of succulents, *Portulaca* is highly adaptable to abiotic stresses like drought, heat, and salinity due to unique physiological mechanisms, such as C_4_ and Crassulacean acid metabolism (CAM) photosynthesis. Its versatility extends to various applications, including soil conditioning, livestock feed, phytoremediation, and pest management. Furthermore, *Portulaca*’s ability to hyperaccumulate heavy metals underscores its potential in environmental cleanup. However, challenges, such as low bioactive compound yields, scalability issues, and regulatory considerations, hinder its broader applications. Advances in metabolomics, genomics, and sustainable cultivation practices are pivotal to unlocking the full potential of *Portulaca* in agriculture, medicine, and environmental sustainability. This review highlights the untapped potential of *Portulaca* in agricultural applications, emphasizing its role in developing climate-resilient crops and innovative therapeutic solutions while also exploring the chemical diversity and biological activities of its bioactive compounds.

## Introduction

*Portulaca*, commonly known as purslane, is a genus of succulent plants in the *Portulacaceae* family. It comprises approximately 100 species, mainly found in tropical and subtropical regions (Ferraz et al. 2024), with several species also occurring in temperate regions across Europe, Canada, the United States, Australia, and New Zealand (Iranshahy et al. 2017). The genus continues to expand taxonomically, as demonstrated by the recent discovery of *Portulaca laljii* in India, a species distinguished by unique traits, such as tuberous roots and trifid styles (Sivaramakrishna & Yugandhar 2020).

The name *Portulaca* is believed to be derived from the Latin words *Porto* (to carry) and *lac* (milk), possibly referring to the plant’s milky sap (Kumar et al. 2022). It has also been suggested that the name originates from the Latin word portula, meaning “little door,” which may refer to the distinctive dehiscence of the fruit, splitting open like a small door to release seeds. Morphologically, *Portulaca* species are characterized by fleshy leaves, thick stems, and small, often sun-sensitive flowers. It displays various inflorescence types, including cymose, capitulate, and solitary forms, with evidence suggesting cymose inflorescences may be ancestral (Ocampo & Mair-Sánchez 2018). Several *Portulaca* species are valued for their horticultural appeal and ecological resilience. *Portulaca grandiflora* and *Portulaca umbraticola* are cultivated for their vibrant, colorful blooms and drought tolerance, making them popular in ornamental horticulture (Souza et al. 2024; da Silva Souza et al. 2024).

Among the species, *Portulaca oleracea* L. is the most extensively studied and widely distributed. The specific epithet *oleracea*, from Latin, means “vegetable” or “herb,” indicating its traditional use as a cultivated edible plant (Obukohwo 2024). Known by various common names around the world, such as purslane in the USA and Australia, rigla in Egypt, pigweed in England, pourpier in France, and Ma-Chi-Xian in China (Baradaran Rahimi et al. 2019), *Portulaca oleracea* L. has been recognized in several national pharmacopeias, such as those of France, Spain, Mexico, and Venezuela (Iranshahy et al. 2017).

*Portulaca oleracea* L. is widely recognized for its superior nutritional profile compared to many leafy vegetables. Edible in both raw and cooked forms, it is a rich source of omega-3 fatty acids, particularly alpha-linolenic acid and gamma-linolenic acid, present at concentrations of 4 mg/g fresh weight (Uddin et al. 2014), as well as essential minerals like potassium, magnesium, calcium, and phosphorus (Srivastava et al. 2021; Nemzer et al. 2021). Its macronutrient composition includes approximately 3% carbohydrate and 2% protein (Srivastava et al. 2021). These attributes make it a promising candidate for addressing micronutrient deficiencies and promoting food security.

Besides its nutritional value, *Portulaca oleracea* L. has long been used in the traditional medicine (Shao et al. 2024). Recognized by the World Health Organization as one of the most widely used medicinal plants, *Portulaca oleracea* L. has earned the title of “Global Panacea” (Li et al. 2024a, b). In traditional Chinese medicine, it is known as the “vegetable for long life” and is used to cool the blood, stop bleeding, and detoxify the body (Mattera et al. 2024). Its aerial parts are prescribed to treat conditions, such as fever, dysentery, diarrhea, carbuncles, eczema, and hematochezia, often in doses of 9–15 g (Li et al. 2013; Zhao et al. 2014). These traditional applications are supported by its phytochemical richness, including alkaloids, flavonoids, phenolic acids, lignans, and betalains (Montoya-García et al. 2023). These bioactive compounds exhibit various beneficial properties, such as antioxidants, anti-inflammatory, antibacterial, and antidiabetic activities, offering potential for use in functional foods and therapeutic applications.

*Portulaca* species also contribute to sustainable agriculture and environmental remediation. Their remarkable adaptability enables them to thrive in environments ranging from arid deserts to humid tropics (Rodrigues Neto et al. 2023). They are particularly tolerance to drought (Ferrari et al. 2020), heat (Ul Haq et al. 2019), and salinity (Borsai et al. 2020). This resilience is attributed to unique physiological mechanisms, such as the facultative ability in some species, such as *Portulaca oleracea* L., to switch between C_4_ and Crassulacean Acid Metabolism (CAM) photosynthesis under drought and salinity stress, thereby optimizing water-use efficiency (Ferrari et al. 2020; Carrascosa et al. 2023). Certain species have shown potential for phytoremediation, effectively accumulating and stabilizing heavy metals in contaminated soils (Alyazouri et al. 2020; Mohammadzadeh & Hajiboland 2022). Moreover, *Portulaca* extracts have demonstrated biopesticidal activity, such as repelling the two-spotted spider mite, and serve as feed additives for livestock (Meteab et al. 2023).

Despite its potential, the commercial adoption of *Portulaca* faces several challenges, such as low yields of bioactive compounds, scalability issues, and regulatory barriers. Overcoming these challenges through sustainable cultivation practices, technological innovation, and supportive policies could unlock its full potential for agriculture, health, and environmental sustainability. While existing reviews have primarily focused on its pharmacological properties or nutritional value, few have synthesized its environmental applications or explored its integration into practical systems. This review addresses that gap by synthesizing up-to-date evidence on *Portulaca*’s abiotic stress tolerance mechanisms and its diverse bioactive profiles. Specifically, it aims to (i) consolidate recent advances in the phytochemistry and pharmacological potential of *Portulaca* species, (ii) highlight their physiological adaptations to salinity, drought, and heavy metal stress, and (iii) explore practical integration strategies for their use as biopesticides, phytoremediators, climate-resilient crops, and animal feed additives. The novelty of this review lies in its interdisciplinary approach, combining metabolomic and genomic insights with field-level applications, to provide a roadmap for harnessing *Portulaca*’s full potential in sustainable agriculture, environmental management, and functional health products. In doing so, this work offers new insights into how *Portulaca* can contribute to climate resilience, low-input agriculture, and circular bioeconomy strategies.

## Global distribution, morphological diversity, and biogeographical evolution

**Fig. 1:**
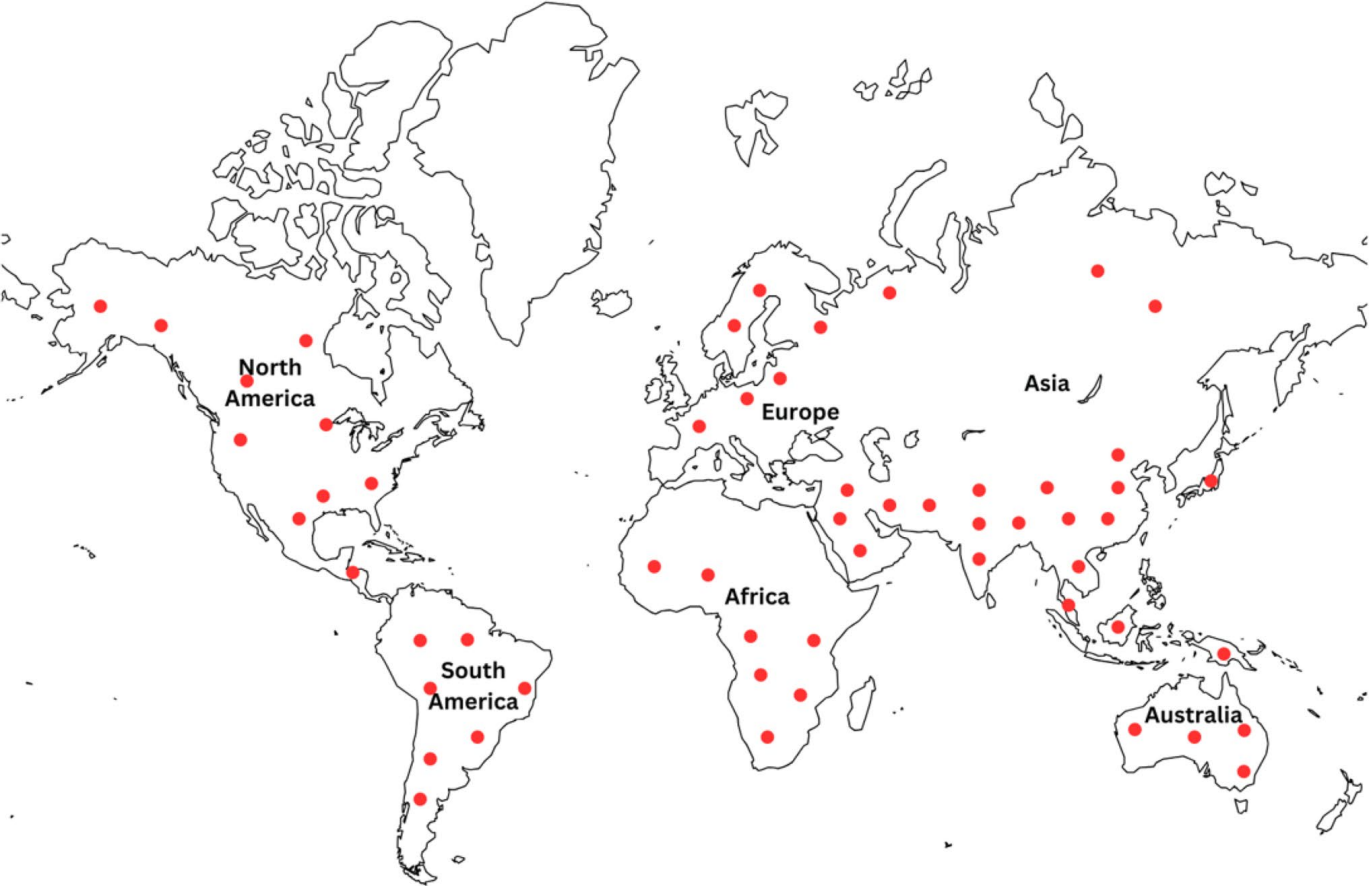
Global distribution of the *genus Portulaca* indicated by red dots on the map

The *Portulaca* genus represents a diverse group of succulent herbaceous plants found worldwide (Srivastava et al. 2023). It grows well in almost all unshaded areas with abundant light exposure (Masoodi et al. 2011). The biogeographical evolution of *Portulaca* can be divided into two major lineages: the opposite-leaved species (OL clade) and alternate-to-subopposite-leaved species (AL clade) (Tamboli et al. 2022). The opposite-leaved species comprises species with opposite leaves, such as *Portulaca hereroensis* and *Portulaca digyna*, primarily distributed across Africa, Asia, and Australia. In contrast, the alternate-to-subopposite-leaved species include species with alternate-to-subopposite leaves, such as *Portulaca oleracea* L. and *Portulaca pilosa* L., which are more widely distributed and predominantly originate from the New World (Ocampo and Columbus 2012). Figure 1 illustrates the global distribution of *Portulaca*, showing its presence across temperate, subtropical, and tropical regions, from sea level up to elevations of 2600 m.

**Fig. 2:**
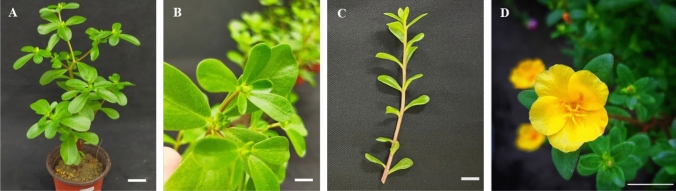
The aerial parts of a *Portulaca* species. **A** Overview of the plant, **B** the apical whorl consisting of four to five oppositely arranged leaves, each measuring approximately 2.0 to 2.5 cm, **C** the opposite leaf arrangement, and **D** the flower of *Portulaca* sp. The stem shows a reddish hue in older sections and a greenish color in younger shoots. The scale bar represents 2 cm

*Portulaca* species can be either annual or perennial prostrate herbs, depending on the climate. For instance, *Portulaca oleracea* L. is typically an annual plant, but it can grow as a perennial in warmer tropical environments such as USDA growing zones 10–11 (Chugh et al. 2019). In colder regions, *Portulaca oleracea* L. behaves as an annual, completing its life cycle before the onset of frost due to its sensitivity to low temperatures and vulnerability to tissue damage (Carrascosa et al. 2023). Some species like *Portulaca juliomartinezii* have been reported as newly identified perennials from central Mexico (Ocampo 2018). Generally, *Portulaca* have stems measuring approximately 15–30 cm in length and around 2 mm in diameter. The stems are either red (due to anthocyanin) or light green and are noticeably swollen at the nodes. The leaves are fleshy, sub-sessile, and typically 2–3 cm long, arranged in an opposite pattern. The petioles are short, typically about 1–1.5 mm long and 0.5 mm thick, with the upper surface being green and the lower surface reddish (Fig. 2).

*Portulaca* flowers bloom when moisture levels are adequate. They are bisexual and measure approximately 0.25–0.50 inches in diameter, with yellow or yellow-tinted petals (typically five, though occasionally four, which are notched at the edges). The stamens and pistils, both yellow, cluster at the center of the flower. *Portulaca* flowers generally open on sunny days, usually from the midmorning to early afternoon. The fruit consists of nearly round to oval capsules, approximately 4–8 mm long, which split open in the middle to release seeds. The root system of *Portulaca* resembles that of most dicotyledonous plants, with a long and thick taproot penetrating deep into the soil accompanied by numerous fibrous lateral roots scattered in the upper soil layers (Kumar and Kaur 2018).

## Bioactive compounds

Several bioactive compounds have been identified across species within the *Portulacaceae* family, with *Portulaca oleracea* L. emerging as one of the most extensively studied due to its rich phytochemical composition. These compounds include flavonoids, alkaloids, phenolics, lignans, betalains, cerebrosides, fatty acids, and terpenoids. Although these bioactivities have prompted increasing interest in other *Portulaca* species, research on species other than *Portulaca oleracea* L. remains limited. This highlights the need for further phytochemical and pharmacological investigations. This section provides an overview of the bioactive compounds found in *Portulaca* species.

### Alkaloids (alkaloids content of fresh purslane)

Alkaloids are a class of low-molecular-weight compounds characterized by heterocyclic nitrogen atoms (Lichman 2021). This group of compounds is predominantly found in plants and has long been of interest in medicine because of its bioactive properties. The ability of some of these compounds to modulate the human central nervous system has been known for decades and has been utilized for pain relief.

To date, more than 70 alkaloids have been identified in various species of the *Portulacaceae* family. The bioactivity of these alkaloids is summarized in Table 1. Among them, several cyclodopa amide alkaloids, such as oleraceins (A, B, C, D, K, N, and O), have been identified in *Portulaca oleracea* L.*, Portulaca rausii*, and *Portulaca granulatostellulata* (Xiang et al. 2005; Jiao et al. 2015). These compounds exhibit antioxidant activities that help reduce oxidative stress in the body, potentially lowering the risk of chronic diseases such as cardiovascular disease and cancer. In addition, oleraceins T, U, V, and W have also been isolated from the same species by Farag and Shakour (2019), but their bioactivities remain unexplored.

**Table 1:** Bioactivities of alkaloids isolated from different *Portulaca* species

Species	Bioactive compounds (alkaloids)	Bioactivities	References
*Portulaca oleracea* L	Aurantiamide	Antioxidant	Chen et al. (2016)
	Aurantiamide acetate	Antioxidant	
	Benzamide	Anticholinesterase	Liu et al. (2021a, b)
*Portulaca oleracea* L.*, Portulaca grandiflora*	Indicaxanthin	Antioxidant	Guerrero-Rubio et al. (2019)
*Portulaca oleracea* L	β-Carboline-3-carboxylic acid	Anticholinesterase	Xiu et al. (2019b)
	(E)-p-Coumaramide	Anticholinesterase	
	(E)-Ferulamide	Anti-inflammation	
	(E)-Ferulamide	Anti-inflammation	
	6,7-Dihydroxy-3,4 dihydroisoquinoline	Anti-inflammation	Jin et al. (2018)
	6,7-Dihydroxy-1-methyl-3,4 dihydroisoquinoline	Anti-inflammation	
*Portulaca oleracea* L.*, Portulaca lutea*	Dopamine	Anti-inflammation	
*Portulaca oleracea* L	(R)-(+)-1-Benzyl-6,7-dihydroxy 1,2,3,4-tetrahydroisoquinoline	Anti-inflammation	
	1-(Furan-2-yl)-6,7-dihydroxy-3,4 dihydroisoquinoline	Anti-inflammation	
	1-(5′-Hydroxylmethylfuran 2-yl)-6,7-dihydrox-y-3,4 dihydroisoquinoline	Anti-inflammation	
	(R)-( +)-1-Isobutyl-6,7-dihydroxy 1,2,3,4-tetrahydroisoquinoline	Anti-inflammation	
	(S)-(−)-Oleracein E	Anti-inflammation	
	Noradrenaline	Neuroprotective	Martins et al. (2016)
	Iseluxine	Antioxidant	Yue et al. (2015)
	Lycoricidine	Cytotoxic	Wei et al. (2019)
*Portulaca oleracea* L.*, Portulaca lutea, Portulaca rausii, Portulaca granulatostellulata*	Oleracein A	Antioxidant	Farag and Shakour (2019), Xiang et al. (2005)
	Oleracein B	Antioxidant	
	Oleracein C	Antioxidant	
	Oleracein D	Antioxidant	
*Portulaca oleracea* L	Oleracein E	Antioxidant, hypoglycemic, antidiabetic, neuroprotective potential	Xiang et al. (2005), Sun et al. (2017), Roozi et al. (2019)
	Oleracein F	Antioxidant	Liu et al. (2011)
	Oleracein G	Antioxidant	
	Oleracein H	Antioxidant	Jiao et al. (2015)
*Portulaca oleracea* L.*, Portulaca rausii, Portulaca granulatostellulata*	Oleracein K	Antioxidant	Jiao et al. (2015), (Farag & Shakour 2019)
	Oleracein N	Antioxidant	
	Oleracein O	Antioxidant	
*Portulaca oleracea* L	Oleracein L	Antioxidant, hypoglycemic, antidiabetic	Jiao et al. (2015), Roozi et al. (2019)
	Oleracein Q	Anti-inflammatory	Zhang et al. (2024)
	Oleracein R	Antioxidant	Jiao et al. (2015)
	Oleracein S	Antioxidant	
*Portulaca oleracea* L.*, Portulaca rausii, Portulaca granulatostellulata*	Oleracein T		Farag and Shakour (2019)
	Oleracein U		
	Oleracein V		
	Oleracein W		
*Portulaca oleracea* L	Oleracein X	Anticholinesterase	Fernandez-Poyatos et al. (2021)
	Oleracein Y	Anticholinesterase	
	Oleracein Z	Anticholinesterase	Fu et al. (2021)
	Oleracein ZA	Anticholinesterase	
	Oleracein ZB	Anticholinesterase	
	Oleraciamide D	Cytotoxic	Zhao et al. (2018)
	Oleraciamide E	Anticholinesterase, antioxidant	Liu et al. (2021a, b)
	Oleraciamide G	Anticholinesterase	Xu et al. (2021)
	Oleracimine C	Anti-inflammatory	Zhang et al. (2025)
	Oleracimine	Anti-inflammatory antioxidant	Li et al. (2016)
	Oleracimine A		
	Oleracone A		
	Oleracone L	Anticholinesterase antioxidant	Cui et al. (2021)
	Oleracone Q	Anti-inflammatory	Zhang et al. (2024)
	Oleracone	Anti-inflammatory	Meng et al. (2016)
	Oleracrylimide A	Anticholinesterase	Song et al. (2022)
	Oleracrylimide B	Anticholinesterase	
	Oleracrylimide C	Anticholinesterase	
	Oleraindole A	Anticholinesterase antioxidant	Zhao et al. (2019a)
	Oleraindole B	Anticholinesterase antioxidant	
	Oleraindole D	Anticholinesterase	Xu et al. (2021)
	Oleraisoindole A	Anticholinesterase	Ma et al. (2021)
	Oleraisoquinoline A	Anti-inflammatory, antioxidant	Zhao et al. (2025)
	Olerapyridine	Anti-inflammatory	Liu et al. (2024)
	Olerapyrimidine	Anti-inflammatory	
	Oleraurea	Anticholinesterase	Xiu et al. (2019b)
	Oxynorchelerythrine	Cytotoxic	Wei et al. (2019)
	Portulacatal	Anticholinesterase anti-inflammatory	Cui et al. (2021)
	Portulacatone	Antioxidant	Yue et al. (2015)
	Portulacatone A	Anti-inflammatory	Meng et al. (2016)
	Portulacatone B	Anticholinesterase anti-inflammatory	Cui et al. (2021)
	Soyalkaloid A	Anticholinesterase, antioxidant	Xiu et al. (2019b), Xiu et al. (2019a)
	(S)-(−)-Salsolinol	Anti-inflammatory	Jin et al. (2018)
	2-Sulfonic acid dopamine	Anti-inflammatory	
	(−)-(R)2-[1-(3,4 Dihydroxyphenethyl)-3-hydroxy-2,5 dioxopyrrolidin-3-yl]acetic acid	Anti-inflammatory	Fu et al. (2021)
	(+)-(S)-2-[1-(3,4 Dihydroxyphenethyl)-3-hydroxy-2,5 dioxopyrrolidin-3-yl]acetic acid	Anti-inflammatory	
	(−)-(R)-2-[1-(4-Hydroxyphenethyl) 3-hydroxy-2,5-dioxopyrrolidin-3-yl] acetic acid	Anti-inflammatory	
	(+)-(S)-2-[1-(4-Hydroxyphenethyl) 3-hydroxy-2,5-dioxopyrrolidin-3-yl] acetic acid	Anti-inflammatory	
	(−)-(S,Z)-5-(3,4 Dihydroxybutylidene) imidazolidi ne-2,4-dione	Anti-inflammatory	
	(2R,3S,3′S,5S,6R)-2-Ethoxy-5 hydroxy-6-(hydroxymethyl) 2′,3′,4′,5,6,9′-hexahydro-2H,4H spiro[pyran-3,1′-pyrido[3,4-b] indole]-3′-carboxylic acid	Anti-inflammatory	
	6,7-Dihydro-1,10,11-trihydroxy benzo[a]-quinolizinium hydroxide inner salt	Anti-inflammatory	
	4-Carboxy-6,7-dihydro-9,10 dihydroxy-benzo-[a]quinolizinium carboxylate inner salt	Anti-inflammatory	
	(−)-(S)-3-Hydroxy-1-(3,4 dihydroxy-phenethyl) pyrrolidine 2,5-dione	Anti-inflammatory	
	(E)-3-[4-(β-D-Glucopyranosyloxy) 3-methyoxyphenyl]-N-[2-(4-hydro xy-3,5-dimet-hoxyphenyl)ethyl]-2 propenamide	Anti-inflammatory	
	(E)-N-[3-Hydroxy-4-(β-D glucopyranosyloxy)phenethyl]-3-(4-hydroxyphenyl)acrylamide	Anti-inflammatory	
	(E)-N-[4-(β-D-Glucopyranosyloxy) phenethyl]-3-(4-hydroxyphenyl) acrylamide	Anti-inflammatory	
	(1S,3S)-1-Methyl-1,2,3,4 tetrahydro-β-carboline-3-carboxylic acid	Anticholinesterase	Xiu et al. (2019b)
	2,3,4,9-Tetrahydro-1H pyrido[3,4-b] indole-3-carboxylic acid	Anticholinesterase	
	6,11-Dihydroxy-8,9 dimethoxybenzo[1,3]-dioxolo[4,5-c] phenanthridin-5(4H)-one	Cytotoxic	Wei et al. (2019)
	6,9,11-Trihydroxybenzo[1,3] dioxolo[4,5-c]phenanthridin-5(4H) one	Cytotoxic	
	1,5-Dimethyl-6-phenyl-1,2-dihydro 1,2,4-triazin-3(2H)-one	Cytotoxic	Tian et al. (2014)
	N-trans-Feruloyl tyramine	Cytotoxic	
	(7′R)-N-Feruloyl normetanephrine	Cytotoxic	
	(3R)-3,5-bis(3-methoxy-4-hydroxyphenyl)-2,3- dihydro-2(1H)-pyridinone	Cytotoxic	
	1,5-dimethyl-6-phenyl-1,2-dihydro-1,2,4- triazin-3(2H)-one	Cytotoxic	

Research to date has mainly focused on *Portulaca oleracea* L., which has yielded the most diverse set of oleraceins. For instance, oleraceins (E, F, G, H, I, K, L, P, Q, R, and S) have only been discovered in *Portulaca oleracea* L. Among these compounds, oleraceins K and L demonstrated the strongest free radical 2,2-diphenyl-1-picrylhydrazyl (DPPH) scavenging activity (Jiao et al. 2015) and exhibited significantly higher antioxidant activities than vitamin C (Xiang et al. 2005; Liu et al. 2011). In addition, oleracein (X, Y, Z, ZA, and ZB) (Fernandez-Poyatos et al. 2021; Fu et al. 2021), oleraciamide (D, E, and G) (Xu et al. 2021; Liu et al. 2021a, b), oleracone L (Cui et al. 2021), oleracrylimide (A, B, and C) (Song et al. 2022), oleraindole (A, B, and D) (Zhao et al. 2019a; Xu et al. 2021), oleraisonidole A (Ma et al. 2021), oleraurea (Xiu et al. 2019a), portulacatal (Cui et al. 2021), portulacatone B (Cui et al. 2021), and soyalkaloid A (Xiu et al. 2019a) have been isolated from *Portulaca oleracea* L. Notably, all of these compounds have demonstrated anticholinesterase activity, suggesting their potential as cholinesterase inhibitors in treating neurodegenerative diseases, such as Alzheimer’s disease.

Several alkaloids with anti-inflammatory properties have been isolated from *Portulaca oleracea* L. These include (−)-(R)2-[1-(3,4 dihydroxyphenethyl)-3-hydroxy-2,5 dioxopyrrolidin-3-yl]acetic acid, (−)-(R)-2-[1-(4-hydroxyphenethyl) 3-hydroxy-2,5-dioxopyrrolidin-3-yl] acetic acid and 6,7-dihydro-1,10,11-trihydroxy benzo[a]-quinolizinium hydroxide inner salt (Fu et al. 2021). Recently, a new alkaloid with antioxidant and anti-inflammatory activity named oleraisoquinoline A was successfully isolated and characterized by Zhao et al. (2025) from *Portulaca oleracea* L. Apart from that, four new alkaloids with anti-inflammatory activity, namely, olerapyrimidine (Liu et al. 2024), olerapyridine (Liu et al. 2024), oleraciamide H (Zhang et al. 2024), and oleracone Q (Zhang et al. 2024), were isolated from *Portulaca oleracea* L. These compounds were reported to significantly inhibit the release of IL-1*β* and TNF-*α* in the RAW 264.7 cells induced by LPS (Zhang et al. 2024; Liu et al. 2024). Other alkaloids, such as oleraciamide D (Xu et al. 2021), 6,9,11-trihydroxybenzo[1,3] dioxolo[4,5-c]phenanthridin-5(4H) one (Wei et al. 2019), N-trans-feruloyl tyramine, (7′R)-N-feruloyl normetanephrine, and 1,5-dimethyl-6-phenyl-1,2-dihydro-1,2,4-triazin-3(2H)-one (Tian et al. 2014), have also been successfully characterized from *Portulaca oleracea* L. and have been reported to demonstrate cytotoxic activity against SH-SY5Y cancer cell lines (Zhao et al. 2018) and A549 human lung cancer cell lines (Wei et al. 2019).

### Flavonoids

Flavonoids are a diverse group of polyphenolic secondary metabolites that consist of two phenyl rings connected to a heterocyclic ring or a C6–C3–C6 carbon structure (Karlson et al. 2022). Flavonoids are classified into several subgroups based on their structure and biosynthesis, including flavones, flavonols, flavanones, flavan-3-ols, isoflavones, and anthocyanins. Over 20 flavonoids have been isolated from the *Portulacaceae* family and reported to possess several bioactivities, such as antioxidant, anti-inflammatory, antibacterial, anticancer, antidiabetic, anti-adipogenesis, and anticholinesterase activities. These bioactivities are attributed to the presence of several flavonoid compounds, such as apigenin, kaempferol, luteolin, and quercetin that are most commonly found in the *Portulacaceae* family including species like *Portulaca oleracea* L. (Xu et al. 2006),* Portulaca lutea* (Mishra et al. 2024), and* Portulaca pilosa* L. (Gatea et al. 2017).

Quercetin exhibits a wide range of bioactivities, including antiphlogistic (Marcolin et al. 2012), anti-asthmatic (Khazdair et al. 2021), anti-adipogenic (Gnoni et al. 2022), antioxidant, anti-tyrosinase, and anti-alpha-glucosidase (Chen et al. 2022). Its anti-adipogenic effects have been shown to effectively reduce lipid accumulation in steatotic cells, suggesting its potential use in treating non-alcoholic fatty liver disease (NAFLD) (Gnoni et al. 2022). Besides, Nayaka et al. (2014) demonstrated that apigenin isolated from *Portulaca oleracea* L. exhibited antibacterial activity against *Pseudomonas aeruginosa*, *Salmonella typhimurium*, *Proteus mirabilis*, *Klebsiella pneumoniae*, and *Enterobacter aerogenes*, highlighting its potential as an antibacterial drug for treating infections caused by these bacteria.

In *Portulaca oleracea* L., several oleracone compounds have been identified, including oleracone C, D, E, F, and G, which have demonstrated various bioactivities. Duan et al. (2022) characterized two new oleracones, J and K, and found that all oleracones exhibited antioxidant and anticholinesterase activities, except oleracone F and oleracone G, which exhibit antioxidant and anti-inflammatory activities (Duan et al. 2022).

Portulacanones (A, B, C, and D), a group of homoisoflavonoids, were the first compounds identified in *Portulaca oleracea* L. and have been reported to exhibit cytotoxic activity against NCI-H460 cell lines (Yan et al. 2012). Other flavonoids identified from *Portulaca oleracea* L.*,* such as HM-chromanone (Park and Han 2022; Park et al. 2019) and myricetin (Liu et al. 2007), also possess antidiabetic or hypoglycemic properties*.* Liu et al. (2007) showed that myricetin improved insulin resistance in rats exhibiting insulin resistance by enhancing insulin action on IRS-1-associated PI 3-kinase and GLUT4 activity. Thus, suggesting it as a promising compound for diabetes treatment.

A summary of the flavonoids isolated from various species within the *Portulacaceae* family, along with their associated bioactivities, is summarized in Table 2.

**Table 2:** Bioactivities of flavonoids isolated from different *Portulaca* species

Species	Bioactive compounds (flavonoids)	Bioactivities	References
*Portulaca oleracea* L.*, Portulaca grandiflora, Portulaca lutea, Portulaca rausii, Portulaca granulatostellulata*	Apigenin	Antibacterial	Nayaka et al. (2014), Farag and Shakour (2019), Mishra et al. (2024), Xu et al. (2006), Gök et al. (2022)
*Portulaca oleracea* L.*, Portulaca pilosa* L.*, Portulaca lutea*	Genistein	Antioxidant, Anti-inflammatory	Gatea et al. (2017), Susutlertpanya et al. (2015), Ji et al. (2011)
*Portulaca oleracea* L	2,2’-Dihydroxy-4’,6’ dimethoxychalcone	Cytotoxic	Yan et al. (2012)
	HM-chromanone	Anti-diabetic, Anti-inflammation, Anti-adipogenesis	Park et al. (2019), Kang et al. (2021), Je et al. (2021), Park and Han (2022)
*Portulaca oleracea* L.*, Portulaca grandiflora, Portulaca pilosa* L.*, Portulaca lutea*	Kaempferol	Antioxidant, anti- asthmatic, anti-tyrosinase, anti-alpha-glucosidase	Xu et al. (2006), Khazdair et al. (2021), Costa et al. (2021), Gatea et al. (2017), Chen et al. (2022)
*Portulaca oleracea* L.*, Portulaca grandiflora, Portulaca pilosa* L.*, Portulaca lutea, Portulaca rausii, Portulaca granulatostellulata*	Luteolin	Anti-inflammatory	Xu et al. (2006), Costa et al. (2021), Gatea et al. (2017), Abu-Elsaad and El-Karef (2019)
*Portulaca oleracea* L.*, Portulaca grandiflora*	Myricetin	Antioxidant, anti-inflammatory, antifibrotic, antiobesity, antidiabetic	Xu et al. (2006), Liu et al. (2007), Semwal et al. (2016), Geng et al. (2017), Guo et al. (2015), Guo et al. (2019), Domitrovic et al. (2015), Hu et al. (2018), Xia et al. (2016), Anghel et al. (2013)
*Portulaca oleracea* L	Oleracone C	Antioxidant, anticholinesterase	Yang et al. (2018)
	Oleracone D	Antioxidant, anticholinesterase	
	Oleracone E	Antioxidant, anticholinesterase	
	Oleracone F	Antioxidant, anti- inflammatory	
	Oleracone G	Antioxidant, anti- inflammatory	Duan et al. (2021)
	Oleracone J	Antioxidant, anticholinesterase	
	Oleracone K	Antioxidant, anticholinesterase	
	Portulacanone A	Cytotoxic, anti- inflammatory	Yan et al. (2012)
	Portulacanone B	Cytotoxic, anti- inflammatory	
	Portulacanone C	Cytotoxic, anti- inflammatory, anti-adipogenic	Yan et al. (2012), Lee et al. (2019)
	Portulacanone D	Cytotoxic, anti- inflammatory	Yan et al. (2012)
*Portulaca oleracea* L.*, Portulaca grandiflora, Portulaca pilosa* L.*, Portulaca lutea*	Quercetin	Antioxidant, anti- asthmatic, anti-adipogenic, anti-inflammatory, anti-tyrosinase, and anti-alpha-glucosidase	Xu et al. (2006), Khazdair et al. (2021), Gatea et al. (2017), Gnoni et al. (2022), Marcolin et al. (2012), Chen et al. (2022)

### Phenolic acids

Phenolic acids are a group of organic compounds that are characterized by a carboxyl group attached to phenol. These compounds have gained a significant attention in human health research owing to their potent anticancer (Kumar et al. 2019), anti-inflammatory (Badhani et al. 2015), and antibacterial properties (Kang et al. 2008). Most of the phenolic acids, including caffeic acid (Zhu et al. 2012), chlorogenic acid (Erkan 2012), ferulic acid (Cheng et al. 2012), rosmarinic acid, and *p*-coumaric acid (Chen et al. 2022), have been identified in *Portulaca oleracea* L.*, Portulaca grandiflora* (Spórna-Kucab et al. 2022), and *Portulaca pilosa* L. (Gatea et al. 2017) (Table 3).

**Table 3:** Bioactivities of phenolic acids isolated from different *Portulaca* species

Species	Bioactive compounds (phenolic acids)	Bioactivities	References
*Portulaca oleracea* L.*,* *Portulaca grandiflora*	Caffeic acid	Antioxidant	Santiago-Saenz et al. (2018), Zhu et al. (2012), Hossain et al. (2010)
*Portulaca oleracea* L.*,* *Portulaca pilosa* L.*,* *Portulaca grandiflora*	Chlorogenic acid	Antioxidant	Spórna-Kucab et al. (2022), Erkan (2012), Gatea et al. (2017)
*Portulaca oleracea* L.*,* *Portulaca grandiflora*	Benzoic acid	Antibacterial	Spórna-Kucab et al. (2022)
*Portulaca oleracea* L.*,* *Portulaca pilosa* L	Cinnamic acid		Gatea et al. (2017)
*Portulaca oleracea* L.*,* *Portulaca grandiflora, Portulaca pilosa* L	Ferulic acid	Antioxidant	Spórna-Kucab et al. (2022), Zhang et al. (2022), Gatea et al. (2017), Erkan (2012)
*Portulaca oleracea* L.*,* *Portulaca grandiflora*	Rosmarinic acid	Antioxidant, anti-tyrosinase, and anti-glucosidase	Spórna-Kucab et al. (2022), Chen et al. (2022)
*Portulaca oleracea* L.*,* *Portulaca grandiflora, Portulaca pilosa* L	*p*-coumaric acid	Antioxidant	Spórna-Kucab et al. (2022), Cheng et al. (2012), Zhang et al. (2022), Gatea et al. (2017), Erkan (2012)
*Portulaca oleracea* L.*,* *Portulaca grandiflora*	Vanillic acid	Antibacterial	Spórna-Kucab et al. (2022), Xu et al. (2017)
*Portulaca grandiflora*	Malic acid	Antioxidant, antibacterial	Spórna-Kucab et al. (2022)
	Feruloylquinic acid	Antioxidant	
	Gluconic acid	Antibacterial	
	Carsonic acid	Antibacterial	
*Portulaca oleracea* L.*,* *Portulaca quadrifida* L	2-Methoxy-4-vinylphenol	Antioxidant, antimicrobial, anti-inflammatory	Saxena and Rao (2021)
	Fumaric acid	Antibacterial, antifungal	Saxena and Rao (2021), Unver (2024)
*Portulaca oleracea* L	Olerabenzofuran	Anti-inflammatory	Tian et al. (2022)
	Oleraindenone		

Ferulic acid, *p*-coumaric acid, and chlorogenic acid have demonstrated varying levels of antioxidant activity (Erkan 2012). In addition to its antioxidant activity, rosmarinic acid found in *Portulaca oleracea* L. (Chen et al. 2022) and *Portulaca grandiflora* (Spórna-Kucab et al. 2022) has also been reported to have anti-tyrosinase and anti-glucosidase activities. Its anti-tyrosinase activity suggests its potential as a therapeutic agent for managing hyperpigmentation disorders (Zaidi et al. 2019). Meanwhile, its anti-alpha-glucosidase activity is significant as alpha-glucosidase is a key enzyme that breaks down carbohydrates into glucose in the gastrointestinal tract. As reported by Bhandariet al. (2018), the inhibition of this enzyme is closely associated with antidiabetic effects. Therefore, rosmarinic acid has emerged as a potential candidate for the management of type 2 diabetes mellitus and for relieving postprandial hyperglycemia.

A recent year’s study conducted by Spórna-Kucab et al. (2022) assessed the antibacterial and antifungal activity of some of the phenolic compound isolated from *Portulaca grandiflora,* including benzoic acid, vanillic acid, malic acid, gluconic acid, carsonic acid, and 2-methoxy-4-vinylphenol against *Staphylococcus aureus, Staphylococcus epidermidis, Micrococcus luteus*, *Bacillus subtilis*, *Bacillus cereus*, *Escherichia coli*, *Salmonella typhimurium*, *Pseudomonas aeruginosa*, *Candida albicans*, *Candida glabrata*, and *Candida krusei.* The findings of this study show that all these compounds possess antimicrobial properties against certain tested bacteria and fungi. Additionally, fumaric acid isolated from *Portulaca oleracea* L. and *Portulaca quadrifida* L. was reported to be effective in inhibiting the growth of *K. pneumoniae*, *E. coli*, *P. aeruginosa*, *and E. aerogenes* with minimal inhibitory concentration (MIC) of 150 µg/mL and 75 µg/mL against *S. aureus* (Unver 2024). In the study, the compound was also tested against several fungi species and the result showed that it has modest antifungal activity against *Candida tropicalis*, *Candida krusei*, *Candida albicans*, *Candida glabrata*, and *Candida parapsilosis* with MIC values of 9.375, 37.5, 4.687, 4.687, and 1.172 mg/mL, respectively. These two studies have demonstrated the potential of these phenolic compounds to be used as antibacterial and antifungal agents in the pharmacotherapeutic field in the future, but further research is still needed to understand their potential toxicity and clinical trials are also essential to validate their efficacy and safety before they can be considered for therapeutic applications.

### Terpenoids

Terpenoids, also known as isoprenoids, represent a large and diverse class of secondary metabolites derived from isoprene units (C₅H₈). They are widely recognized for their pharmacological relevance, exhibiting a broad spectrum of bioactivities, including antioxidant, anti-inflammatory, antimicrobial, and anticancer effects. Despite their established significance in plant-based therapeutics, the terpenoid profile of the *Portulaca* genus remains relatively underexplored compared to other medicinally important plant taxa.

Among the limited number of terpenoids identified in *Portulaca* species, two bioactive compounds, lupeol and taraxerol, have been reported from *Portulaca oleracea* L., *Portulaca grandiflora*, and *Portulaca quadrifida* L. (Elkhayat et al. 2008; Mir and Ali 2016; Mus’hib and Abdul-jalil 2024; Saxena and Rao 2021). Lupeol, a pentacyclic triterpenoid, has attracted a significant attention due to its wide-ranging biological activities, including antioxidant, anti-neuroinflammatory, anti-inflammatory, anticancer, and antimicrobial properties (Park et al. 2023). Several studies have demonstrated the therapeutic potential of lupeol. For instance, Beserra et al. (2019) showed that lupeol significantly promoted wound healing in streptozotocin-induced hyperglycemic rats by enhancing the expression of antioxidant enzymes, such as superoxide dismutase 2 (*SOD-2*) and heme oxygenase-1 (*HO-1*). Additionally, lupeol treatment reduced the expression of pro-inflammatory cytokines (e.g., IL-6) while upregulating anti-inflammatory cytokines like IL-10, suggesting its potential utility in treating chronic diabetic wounds (Beserra et al. 2019). Similarly, Ahmad et al. (2020) reported that lupeol reduced Alzheimer’s disease pathology in an Aβ_1–42_-induced mouse model by reducing β-amyloid accumulation and downregulating beta-secretase-1 (*BACE-1*) expression. The study further showed that lupeol inhibited neuroinflammation through suppression of activated glial cells and inflammatory mediators. Besides, lupeol has also demonstrated cytotoxic activity against various cancer cell lines, including human lung carcinoma (H1299) (Park et al. 2020) and osteosarcoma (U-2 OS) (Hsu et al. 2019), as reviewed by Liu et al. (2021a, b).

Taraxerol, another triterpenoid identified in *Portulaca*, also exhibits promising pharmacological properties. A study by Mongalo et al. (2022) showed that both lupeol and taraxerol had significant anti-inflammatory activity, outperforming quercetin (a standard control) in 15-lipoxygenase (15-LOX) inhibition and nitric oxide suppression assays using RAW264.7 macrophage cells. Notably, lupeol exhibited stronger antibacterial activity than vancomycin against *Mycobacterium smegmatis*, *Mycobacterium hominis*, and *Escherichia coli*, with a minimum inhibitory concentration (MIC) as low as 10 µg/mL. Meanwhile, taraxerol demonstrated broad-spectrum antimicrobial activity against pathogens including *Staphylococcus aureus*, *Bacillus subtilis*, *Klebsiella pneumoniae*, *Candida albicans*, and *Aspergillus niger* (Mus et al. 2022). Although these bioactivities were not assessed directly using *Portulaca*-derived lupeol, the pharmacological relevance of these compounds supports the idea that *Portulaca* species could serve as valuable natural sources of medicinal terpenoids. Further phytochemical and pharmacological investigations are needed to fully elucidate the therapeutic potential of terpenoids within this genus.

### Lignans

Lignans are polyphenolic compounds composed of two phenylpropanoid units (C_6_–C_3_) connected by a bond, typically in a biphenyl or dihydroxyphenylpropanoid arrangement. To date, most lignans have been isolated from *Portulaca oleracea* L. with limited reports on their isolation from other species in the *Portulacaceae* family. Three lignans with antioxidant properties, oleralignan, (+)-lirioresinol A, and (+)-syringaresinol, were isolated from *Portulaca oleracea* L. (Ma et al. 2020). Duan et al. (2021) identified the presence of oleralignan B in *Portulaca oleracea* L. and demonstrated its antioxidant and anti-inflammatory activities*.*

Recently, four new lignans named oleralignan A (Xu et al. 2022), oleralignan B (Duan et al. 2021), and oleralignan C and D (Wang et al. 2022a) were also identified in the same species. Among them, oleralignan A was evaluated for its anticholinesterase activity using a microplate assay and exhibited enzyme inhibition at 58.31 ± 0.23 μΜ (Xu et al. 2022). This finding highlights the potential of oleralignan A as a candidate for managing health conditions associated with acetylcholinesterase activity, such as Alzheimer's disease. All the lignans isolated from various species within the *Portulacaceae* family and their bioactivities are summarized in Table 4.

**Table 4:** Bioactivities of different compound groups isolated from various *Portulaca* species

Species	Bioactive compounds	Bioactivities	References
**Terpenoids**			
*Portulaca oleracea* L.*, Portulaca grandiflora, Portulaca quadrifida* L	Lupeol	Antioxidant, anti-neuroinflammatory, anti-inflammatory, cytotoxic	Elkhayat et al. (2008), Mus’hib and Abdul-jalil (2024), Saxena and Rao (2021), Park et al. (2023), Tiwari et al. (2021), Liu et al. (2021a, b), Gallo and Sarachine (2009)
*Portulaca oleracea* L	Taraxerol	Antioxidant, antimicrobial, anti-inflammatory	Mir and Ali (2016), Mus et al. (2022)
**Lignans**			
*Portulaca oleracea* L	Monomethyl-3,3’,4,4’-tetrahydroxy-δ truxinate	Antioxidant	Ma et al. (2020)
	Oleralignan		
	(+)-lirioresinol A		
	(+)-syringaresinol		
	Oleralignan A	Anticholinesterase	Xu et al. (2022)
	Oleralignan B	Antioxidant, anti-inflammatory	Duan et al. (2021)
	Oleralignan C	Antioxidant	Wang et al. (2022a)
	Oleralignan D		
**Cerebrosides**			
*Portulaca oleracea* L	Portulacerebroside A	Cytotoxic	Ji et al. (2015)
	Portulacerebroside B	Antibacterial	Lei et al. (2015)
	Portulacerebroside C		
	Portulacerebroside D		
**Betalains**			
*Portulaca grandiflora*	Vulgaxanthin I	Antioxidant, anti-inflammatory	Guerrero-Rubio et al. (2019), Wang et al. (2022b)
*Portulaca oleracea* L.*,* *Portulaca grandiflora*	Indicaxanthin	Antioxidant, anti-inflammatory	
*Portulaca grandiflora*	Dopaxanthin	Antibacterial, antifungal	Gandía-Herrero et al. (2005), Spórna-Kucab et al. (2022)
*Portulaca oleracea* L.*,* *Portulaca grandiflora*	Betanin	Antioxidant, anti-inflammatory, cognitive improvement, neuroprotective, antibacterial	Esatbeyoglu et al. (2014), Wang et al. (2022b), Esatbeyoglu et al. (2015), Wang and Yang (2010), Spórna-Kucab et al. (2022)
**Fatty acids**			
*Portulaca oleracea* L.*, Portulaca rausii*, *Portulaca granulatostellulata*	Alpha-linolenic acid (ALA)	Decrease omega-6/omega-3 ratio	Farag and Shakour (2019), Uddin et al. (2014)
*Portulaca oleracea* L	Gamma-linolenic acid	Decrease omega-6/omega-3 ratio, anti-inflammatory	Uddin et al. (2014), Rengachar et al. (2022), Kapoor and Huag (2006)
	Docosahexaenoic acid		Uddin et al. (2014)
	Docosapentaenoic acid	Decrease omega-6/omega-3 ratio	
*Portulaca oleracea* L.*, Portulaca lutea, Portulaca rausii, Portulaca granulatostellulata*	Eicosapentaenoic acid	Decrease omega-6/omega-3 ratio, anti-inflammatory, antioxidant	Uddin et al. (2014), Tanaka et al. (2008)
*Portulaca quadrifida* L	Eicosanoic acid	α-Glucosidase inhibitor	Saxena and Rao (2021)
	Hexadecanoic acid	Antioxidant, antimicrobial, hypocholesterolenic, anti-androgenic, 5-α reductase inhibitor	Saxena and Rao (2021)
	n-hexadecanoic acid	Anticancer, antioxidant	Saxena and Rao (2021)
*Portulaca oleracea* L.*, Portulaca quadrifida* L	Octadecanoic acid	Anti-inflammatory, hypocholesterolemic, anticancer, hepatoprotective, antieczemic, nematicide, 5-α reductase inhibitor, anticoronary, antiarthritic, antipsychotic, antibacterial	Saxena and Rao (2021)
*Portulaca oleracea* L.*, Portulaca rausii, Portulaca granulatostellulata*	Myristoleic acid	Reduce risk of cardiovascular disease	Farag and Shakour (2019), Petropoulos et al. (2015)
	Ricinoleic acid		
**Polysaccharides**			
*Portulaca oleracea* L	*Portulaca oleracea* L. polysaccharide (POP or POLP)	Antioxidant, anti-inflammatory, immunomodulatory, antimicrobial, anticancer, neuroprotective, gastroprotective, regulate gut microbiota, metabolic effect, antifatigue, antidiabetic, hypolipidemic, anti-tumor, anticancer, anti-colitis, anti-lead poisoning	Xu and Shan (2014), Hu et al. (2019), Tao et al. (2018), Georgiev et al. (2017), Bai et al. (2016), Zhao et al. (2017), Jia et al. (2021), Li et al. (2014a), Hu et al. (2019), Yang et al. (2024), Chen et al. (2011), Jia et al. (2021), Zhao et al. (2017)
	Crude *Portulaca Oleracea* L. polysaccharide (CPOP)	Anti-diabetic	Bai et al. (2016)
	Water-soluble polysaccharide (POL-P3b)	Antitumor, anticancer, immunomodulatory	Xie et al. (2020), Shen et al. (2013), Zhao et al. (2019b), Jia et al. (2021), Zhao et al. (2013), Zhao et al. (2015)

### Cerebrosides

Cerebrosides are a class of glycosphingolipids that are essential components of the cell membranes of plants and animals. These compounds consist of ceramide backbones linked to a single sugar molecule, typically glucose or galactose. In the *Portulacaceae* family, most cerebrosides have been isolated from *Portulaca oleracea* L., with few reports of their presence in other species within the family (Table 4)*.* Portulacerebrosides (A, B, C, and D) were first isolated from *Portulaca oleracea* L. by Lei et al. (2015) and have demonstrated antibacterial activity against common enteropathogenic bacteria. Among these, portulacerebrosides (B, C, and D) exhibited significant antibacterial effects, revealing their potential for therapeutic use in treating bacillary dysentery. In another study, Ji et al. (2015) investigated the cytotoxic activity of portulacerebroside A in human liver cancer HCCMLM3 cells. The finding showed that this compound inhibited the invasion and metastasis of cancer cells, suggesting its potential as a treatment for liver cancer (Ji et al. 2015).

### Betalains

Betalains are alkaloid-derived pigments predominantly found in plants of the order *Caryophyllales*, including beetroot, amaranth, cactus, and purslane. These compounds can be classified into two groups, betacyanins and betaxanthins. To date, betacyanin and betaxanthin have mostly been identified in *Portulaca oleracea* L. and *Portulaca grandiflora* (Table 4) (Spórna-Kucab et al. 2022).

Betaxanthins identified in *Portulaca grandiflora* include portulacaxanthin II, portulacaxanthin III (Trezzini & Zrÿd 1991), dopaxanthin, vulgaxanthin I, miraxanthin V, and indicaxanthin (Gandía-Herrero et al. 2005). Among these, vulgaxanthin I and indicaxanthin have been shown to possess anti-inflammatory effects (Wang et al. 2022b). Betacyanin isolated from *Portulaca oleracea* L. has been reported to exhibit a more significant effect than vitamin C in ameliorating cognitive deficits in mice owing to its potent antioxidant activities (Wang & Yang 2010). A study conducted by Wang et al. (2022b) further revealed the positive effect of betanin in mitigating oxidative stress and inflammation in human gut cells by suppressing intracellular reactive oxygen species (ROS) production through the activation of Nrf2-signaling and suppression of inflammatory signaling pathways.

Additionally, betaxanthins isolated from *Portulaca grandiflora*, including dopaxanthin, were shown to exhibit antibacterial activity against Gram-positive bacteria (*Staphylococcus aureus*, *Staphylococcus epidermidis*, *Micrococcus luteus*, *Bacillus subtilis*, and *Bacillus cereus*), whereas betacyanin is effective against Gram-negative bacteria (*Escherichia coli*, *Salmonella typhimurium*, and *Pseudomonas aeruginosa*) (Spórna-Kucab et al. 2022). Thus, the antibacterial activity of betaxanthins and betacyanins across a wide range of bacteria underscores their potential for developing promising biocidal agents.

### Fatty acids

Purslane plants are known for their rich fatty acid composition, particularly *Portulaca oleracea* L., which is distinguished by its high omega-3 fatty acid content (0.9 g/100 g), a characteristic not commonly found in plants (Uddin et al. 2014). While omega-3 fatty acids are typically sourced from fish and other animal products, purslane is one of the richest plant-based sources of omega-3 fatty acid that do not contain any cholesterol (Uddin et al. 2014). Nemzer et al. (2021) reported that *Portulaca oleracea* L. contains significantly higher fatty acid and mineral content than spinach and kale, with concentrations of linoleic and alpha-linolenic acids approximately ten times greater. This unique characteristic makes purslanes a valuable addition to plant-based diets, offering a healthier, animal-free alternative to support heart health, reduce inflammation, and promote overall well-being. In addition to *Portulaca oleracea* L., alpha-linolenic acid has also been identified in *Portulaca rausii* and *Portulaca granulatostellulata* (Farag & Shakour 2019).

Eicosapentaenoic acid (EPA) has also been reported in *Portulaca oleracea* L.*, Portulaca lutea, Portulaca rausii*, and *Portulaca granulatostellulata* (Uddin et al. 2014; Farag & Shakour 2019; Mishra et al. 2024), whereas docosahexaenoic acid (DHA) and docosapentaenoic acid (DPA) were found only in *Portulaca oleracea* L. (Uddin et al. 2014). Other two fatty acids, myristoleic acid and ricinoleic acid, were identified in *Portulaca oleracea* L.*, Portulaca lutea*, *Portulaca rausii*, and *Portulaca granulatostellulata* (Farag and Shakour 2019). These two fatty acids are particularly noteworthy for their potential to reduce the risk of cardiovascular disease, as highlighted by Petropoulos et al. (2015). A summary of all fatty acids identified across various species within the *Portulacaceae* family, and their associated bioactivities is provided in Table 4.

### Polysaccharides

Large amounts of polysaccharides are present in *Portulaca oleracea* L., comprising approximately 6.45% of the total weight of the plant (Shen et al. 2013). These polysaccharides exhibit a wide range of bioactivities, including antidiabetic (Hu et al. 2019), immunomodulatory (Georgiev et al. 2017), antioxidant (Li et al. 2014a), neuroprotective (Bai et al. 2016), and anti-inflammatory effects (Yang et al. 2024) (Table 4).

Previous studies have highlighted the anti-tumor effects of *Portulaca oleracea* L. polysaccharide (POP) or water-soluble type of POP (POL-P3b) in various models, including HeLa cells (Zhao et al. 2017), HepG2 cells (Chen et al. 2010), intestinal dendritic cell (Zhao et al. 2019b), gastric cancer (Li et al. 2014b), transplantable sarcoma 180 (Shen et al. 2013), cervical carcinoma, and cervical cancer cells (Zhao et al. 2013). Jia et al. (2021) reported the potential of POL-P3b in enhancing the immune efficacy of dendritic cell vaccines for breast cancer therapy by enhancing specific anti-tumor immune responses via the TLR4/MyD88/NF-κB signaling pathway to induce the activation and maturation of dendritic cells.

POP was also reported by He et al. (2023) to demonstrate strong immune-boosting activity through macrophage activation via the ERK and NF-κB signaling pathways. This finding highlights its potential use as a vaccine adjuvant for infectious and viral diseases. Zhuang et al. (2022) isolated and elucidated the structure of a specific polysaccharide fraction (POP Z) from *Portulaca oleracea* L. The potential of POP Z to relieve lipopolysaccharide-induced inflammatory responses and the intestinal epithelial barrier by inactivating the TLR4/NF-κB pathway while activating the EGF/EGFR pathway was also reported in this study. The findings of this study provide a solid foundation for developing POP as a therapeutic agent for intestinal disease. Another study demonstrated the neuroprotective effect of POP against lead-induced learning and memory impairments in rats (Tao et al. 2018). The neuroprotective activity of POP against lead-induced oxidative toxicity was shown to be related to its antioxidant properties. The study showed that POP treatment could reverse Pb-induced spine loss in the CA1 and DG areas of mouse brains and inhibit lead-induced cognitive impairment. Additionally, Ning et al. (2024) reported that POP could alleviate ulcerative colitis (UC) by regulating intestinal homeostasis.

Overall, these findings collectively underscore POP’s versatility and therapeutic promise in various medical applications. However, research has predominantly focused on polysaccharides from *Portulaca oleracea* L., leaving other species within the *Portulacaceae* family largely unexplored. This highlights the need for further exploration and characterization of polysaccharides from these less-studied species.

## Advances in identifying bioactive compounds

### Metabolomics and analytical techniques

Metabolomics is a powerful approach for investigating metabolic pathways and profiling metabolites in biological systems. Since the accurate identification and quantification of metabolites in *Portulaca* species require sophisticated, highly sensitive analytical techniques, the progression of metabolomic studies heavily relies on advancements in analytical instruments for the exploration of a plant’s chemical composition, with applications ranging from bioactive compound discovery to quality control in herbal products.

Metabolomics can be approached in three primary ways: targeted, semi-targeted, and untargeted (Allwood et al. 2021). Untargeted metabolomics is often used for metabolite identification and quantification with coverage of all detectable metabolites in the sample, whereas targeted metabolomics focuses on specific metabolites or a set of known metabolites (Beger et al. 2024). Semi-targeted metabolomics combines both approaches, analyzing a predefined set of metabolites while remaining open to the identification of other unexpected compounds (Billet et al. 2018). This approach might be useful in *Portulaca* studies aimed at profiling specific bioactive compounds like alkaloids, flavonoids, and fatty acids.

The choice of analytical technique depends on the nature of the metabolites of interest, with various technologies available to researchers. Mass spectrometry (MS)-based methods, such as gas chromatography–mass spectrometry (GC–MS), liquid chromatography–mass spectroscopy (LC–MS), and spectroscopic techniques such as 1D and 2D nuclear magnetic resonance (NMR) and circular dichroism (CD), are commonly used for metabolomic analysis of plant extracts (Vitale et al. 2024). These instruments provide detailed chemical information, such as molecular weight, structure, and fragmentation patterns. The MS-based methods are often combined with spectroscopic techniques to elucidate the structures of the detected compounds (Letertre et al. 2020). The advantage of this hybrid technique is that while the MS-based method provides detailed information on molecular weight, elemental composition, and fragmentation patterns, the spectroscopic techniques offer insights into the structural framework, functional groups, and stereochemistry of metabolites (Scognamiglio et al. 2015). Therefore, this hybrid technique has become the most preferred method for compound identification in plant metabolomics.

Additionally, newer innovations have led to instruments with enhanced sensitivity and resolution, allowing for the detection of low-abundance metabolites crucial for identifying *Portulaca* species’ pharmacological properties. For instance, the triple-quadrupole mass spectrometry (QqQ) and high-resolution MS (HR-MS) instruments can detect trace metabolites with high sensitivity. However, it is essential to note that HR-MS often faces limitations in differentiating and identifying co-eluting isomeric and isobaric compounds due to their identical or nearly identical mass-to-charge (m/z) ratios (Gaudêncio et al. 2023). Therefore, additional separation techniques or complementary structural elucidation methods, such as tandem MS (MS/MS) or NMR, are usually required to resolve these compounds (Helies et al. 2021). The use of ultra-high-performance liquid chromatography coupled with triple-quadrupole mass spectrometry (UHPLC-QqQ) was highlighted in research conducted by Hu et al. (2021), in which 43 compounds, including protocatechuic acid, puerarin, acetoside, and daidzin were successfully quantified from herbal spirits. Besides, mass analyzers, such as TOF (Time-of-Flight), Q-TOF (Quadrupole-Time-of-Flight), and LTQ (Linear Trap Quadrupole)-Orbitrap, are also often incorporated in many different hybrid detection systems, especially in HR-MS/MS due to their ability to remarkably increase the informative power of the MS detectors (Gaudêncio et al. 2023). For example, Xu et al. (2021) successfully isolated seven bioactive compounds, including a novel lignan named oleralignan A, by utilizing HR-MS coupled with ESI and TOF. Ultra-high-performance liquid chromatography electrospray ionization quadrupole time-of-flight mass spectrometry (UHPLC-ESI-Q-TOF–MS) is another state-of-the-art technique that combines ultra-high-performance liquid chromatography with electrospray ionization and quadrupole time-of-flight mass spectrometry. This powerful tool has been widely employed in combination with NMR in recent studies to isolate and characterize bioactive compounds like oleracone L, portulacatone B, portulalcatal (Cui et al. 2021), oleraindenone, olerabenzofuran (Tian et al. 2022), oleraisoindole B (Yao et al. 2023), and oleraisoquinoline A (Zhao et al. 2025) from *Portulaca oleracea* L. In addition, a new alkaloid with anti-inflammatory activity named oleracimine C was successfully isolated and characterized by Zhang et al. (2025) using UHPLC-ESI-Q-TOF–MS, NMR and CD methods. Similarly, high-resolution electrospray ionization time-of-flight mass spectrometry (HR-ESI-TOF–MS) was instrumental in isolating metabolites and other compounds like oleraciamide G and oleraindole D from *Portulaca oleracea* L. (Xu et al. 2021), further underscoring its value in discovering bioactive molecules.

Other advanced mass spectrometry-based techniques, such as matrix-assisted laser desorption/ionization mass spectrometry imaging (MSI) and ion mobility spectrometry (IMS), are also powerful tools for analyzing the spatial distribution and structural characteristics of compounds in complex samples.

### Genomics techniques

Advances in genomics, particularly through whole-genome sequencing (WGS), have revolutionized the study of plant specialized metabolism by enabling comprehensive access to genetic blueprints. These technologies allow identifying biosynthetic genes and biosynthetic gene clusters (BGCs) through genome mining approaches. Such approaches might offer significant potential for uncovering novel compounds with therapeutic or agricultural relevance in *Portulaca* species.

Although WGS technologies have evolved through three generations, the second-generation sequencing, also known as Next-Generation Sequencing (NGS), remains the most widely used due to its capacity to sequence millions of short DNA fragments rapidly and at relatively low cost. Among these, Illumina technology is notable for its high-throughput and accuracy (Kumar et al. 2024). Third-generation sequencing platforms, including Pacific Biosciences’ single-molecule real-time (SMRT) sequencing (Ardui et al. 2018) and Oxford Nanopore Technologies (ONT) (Lu et al. 2016), can generate much longer reads, up to several kilobases, making them well suited for assembling complex genomes and resolving repetitive regions often missed by short-read methods (Rhoads and Au 2015).

Each platform has distinct advantages and limitations. Illumina short reads provide high accuracy and are ideal for resequencing and transcriptome profiling. However, its limited read length often results in fragmented genome assemblies (Caesar et al. 2021). Conversely, PacBio and ONT generate long reads that can span entire BGCs or large gene families. However, this comes at the expense of higher per-read error rates compared to short-read platforms. To overcome these limitations, hybrid sequencing approaches have become increasingly popular. By combining long-read platforms (e.g., PacBio or ONT) with high-accuracy short-read data (e.g., Illumina), researchers can generate high-quality, contiguous genome assemblies suitable for accurate gene annotation and pathway reconstruction (Kumar et al. 2024; Cheng et al. 2025). For instance, Usha et al. (2022) demonstrated the capability of hybrid sequencing to generate a high-quality *Punica granatum* genome (~ 361.76 Mb, N50 of 40 Mb, 90% completeness). The data generated enable identification of key genes involved in flavonoid and phenylpropanoid biosynthesis. Similarly, in *Portulaca oleracea* L., a chromosome-scale genome was assembled in 2023 using ONT, Illumina, and Hi-C sequencing technologies (Wang et al. 2023). This high-resolution genomic resource, now publicly accessible via the Genome Warehouse (GWH), provides end-to-end chromosomal scaffolding that preserves gene order and continuity (Liu et al. 2025). Such continuity enables more accurate identification and functional analysis of biosynthetic genes and gene clusters, especially in complex or repetitive genomic regions (Totikov et al. 2021).

Besides advances in sequencing technologies, the development of tools, such as PlantiSMASH (Kautsar et al. 2017) and PhytoClust (Töpfer et al. 2017), has significantly improved the identification of BGCs in plant genomes. Using PlantiSMASH, Qiao et al. (2022) identified 27 putative BGCs in *Camellia sinensis* L., including those involved in saccharide, terpene, alkaloid, and polyketide biosynthesis. Similarly, Öz (2024) used PlantiSMASH to predict clusters for polyketide, saccharide-terpene, and alkaloid pathways in *Helianthus annuus*. PhytoClust has also been applied to *Nicotiana tabacum*, where 34 BGCs potentially involved in terpenoid biosynthesis were identified, with two confirmed in capsidiol production (Chen et al. 2019). Notably, the capsidiol-related cluster was found to be conserved in *Nicotiana sylvestris*, *Nicotiana tomentosiformis*, and *Nicotiana attenuata* (Chen et al. 2019). These studies highlight the potential of genome mining tools in elucidating plant biosynthetic pathways and underscore their potential application in *Portulaca* species for the discovery of novel metabolites.

**Fig. 3:**
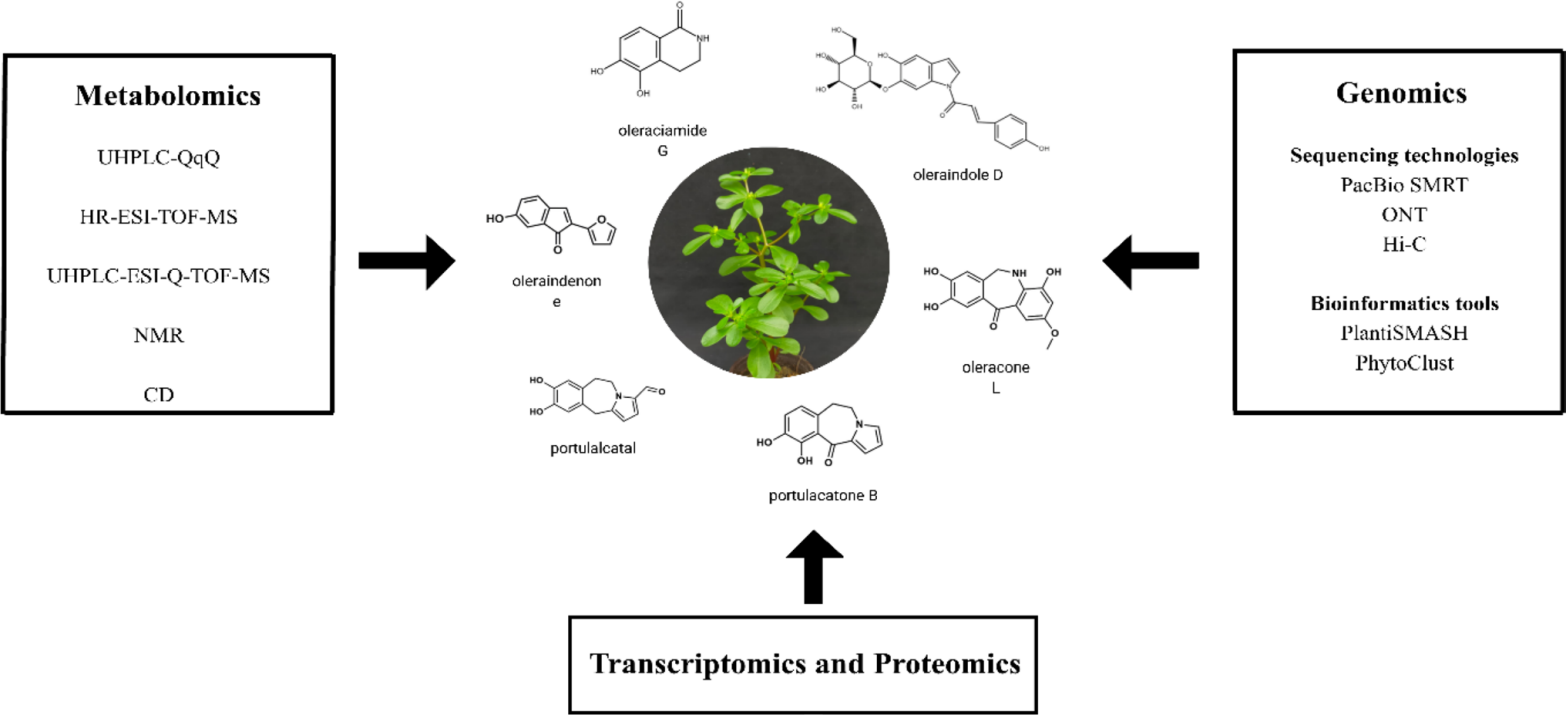
Identification of bioactive compounds in *Portulaca* through integrated omics approaches and bioinformatics analysis

Despite these advances, significant gaps remain in our understanding of *Portulaca* genomics. To date, *Portulaca oleracea* L. is the only species in the genus with a chromosome-scale genome assembly, leaving many other species poorly characterized at the genomic level. Moreover, the regulatory mechanisms governing BGC expression under environmental or developmental cues remain largely unknown. Understanding these regulatory networks is critical, as they directly influence the levels of secondary metabolite production. Furthermore, while genomic sequencing provides a powerful tool for predicting biosynthetic capacity, it does not always reflect actual metabolite production. Gene expression alone may not correlate with the accumulation of secondary metabolites due to complex regulatory pathways, environmental variability, and tissue-specific expression patterns. Therefore, a multi-omics approach that integrates genomics with transcriptomics, metabolomics, and functional assays is essential for validating the presence and activity of identified compounds (Fig. 3). For instance, metabolic profiling techniques such as LC–MS and NMR, as discussed earlier, can be used together with genome mining to detect and quantify metabolites directly in plant extracts. These techniques not only confirm the functionality of predicted pathways but also reveal novel intermediates or end-products not evident from genomic data alone.

## Agricultural applications

The diverse genus *Portulaca* offers a wide array of applications ranging from sustainable agriculture to cosmetic products. This section explores the multifaceted uses of *Portulaca* species, focusing on its role in biopesticides, phytoremediation, and other applications. *Portulaca oleracea* L., in particular, shows promise as a natural insecticide due to its secondary metabolites and excels in heavy metal accumulation for environmental remediation. Additionally, *Portulaca* species are gaining attention in animal feed and for their ornamental and nutritional value, underscoring their economic and ecological importance.

### Biopesticide

In recent decades, biopesticides have garnered increasing attention as sustainable alternatives for pest management. Among the promising candidates for biopesticides are plants from the *Portulacaceae* family, particularly *Portulaca oleracea* L. These plants contain various secondary metabolites, such as alkaloids, phenolics, terpenoids, and saponins, which play a crucial role in the plant’s defense system against pests and other environmental stressors. Many of these compounds have pest-deterrent properties, making them suitable biopesticides. For instance, the alcoholic extract of *Portulaca oleracea* L. has been shown to effectively control two-spotted adult spider mites (*Tetranychus urticae*) on okra plants, achieving a mortality rate of 68.61% (Meteab et al. 2023). Similar results were also reported by Dalia et al. (2023), where the acetone extract of *Portulaca oleracea* L. resulted in mortality rate ranging from 21 to 91% for *Tetranychus urticae,* with minimal effect on the predatory insect *Orius albidipennis*. Another study by Tayyab et al. (2022) investigated the insecticidal potential of 40 indigenous plant species, including *Portulaca oleracea* L., on Asian citrus psyllids (*Diaphorina citri*). The acetone extract of *Portulaca oleracea* L. leaves caused 41.11% mortality in these pests. Similarly, Su et al. (2005) investigated various *Portulaca oleracea* L. extracts for their contact toxicity and anti-feeding toxicity against *Aphis gossypii.* Among the nine extracts tested, the methanol extract exhibited the highest contact toxicity, whereas the dichloromethane extract exhibited the highest anti-feeding toxicity. The presence of cardiac glycosides and oxalic acids in *Portulaca oleracea* L. may be responsible for its toxicity. In a more recent study, Wang et al. (2020a, b) demonstrated the effectiveness of *Portulaca oleracea* L. against *Spodoptera litura*. They found that a combination of portulacanone A and (9Z,11E,15Z)-13-hydroxyoctadeca-9,11,15-trienoic acid exhibited stronger anti-insect activity than other combinations of isolated compounds (Wang et al. 2020a).

To practically integrate *Portulaca oleracea* L. as a biopesticide in agricultural systems, several strategies should be considered. First, developing standardized extraction and formulation methods is essential. Current research employs different types of solvents used (e.g., methanol, acetone, ethanol, and dichloromethane), extraction conditions (e.g., temperature, time, and solvent-to-material ratio), and preparation techniques, making it difficult to compare results or replicate outcomes in real-world settings. For effective field application, it is essential to establish optimized protocols for extracting the most bioactive compounds in a reproducible and scalable manner. Moreover, formulating these extracts into stable, user-friendly products, such as emulsifiable concentrates, nano-formulations, or water-dispersible powders, would improve field usability and in pest control outcomes. Second, incorporating *Portulaca oleracea*-based sprays into integrated pest management (IPM) programs could provide significant benefits, including effective pest control and a reduced reliance on synthetic chemical pesticides. These botanical extracts can be applied during critical pest infestation periods or rotated with synthetic agents to delay resistance development. Moreover, *Portulaca oleracea* L. can be cultivated as a dual-purpose crop, grown along field margins or within intercropping systems. In such setups, it can serve as both a natural ground cover (reducing weed pressure and conserving soil moisture) and a biopesticide source. Extracts can be freshly prepared on-site or sourced from small-scale processing units, offering low-cost solutions for smallholder and organic farmers. Furthermore, due to its tolerance to poor and saline soils, *Portulaca oleracea* L. can be cultivated on marginal lands, promoting sustainable intensification without competing with primary food crops.

These findings highlight the potential of *Portulaca* plants as an effective biopesticide, demonstrating its broad-spectrum insecticidal properties against multiple pest species. However, while these studies show promising results, further research is needed to better understand the optimal extraction methods, concentrations, and application techniques for maximizing efficacy. Additionally, investigating the environmental impact and safety of these biopesticides on non-target species, particularly beneficial insects, should be prioritized to ensure sustainable pest control solutions. As the search for eco-friendly alternatives to chemical pesticides intensifies, *Portulaca* plants could play a significant role in the development of sustainable and less toxic pest management strategies.

### Phytoremediation

Several *Portulaca* species, including *Portulaca oleracea* L.*, Portulaca tuberosa* Roxb., and *Portulaca grandiflora*, have demonstrated strong potential for phytoremediation, likely due to their ability to tolerate and stabilize heavy metal contaminants. *Portulaca oleracea* L., in particular, has been shown to effectively uptake and accumulate various heavy metals, including copper (Cu), nickel (Ni), mercury (Hg), lead (Pb) (Dwivedi et al. 2012), cadmium (Cd), chromium (Cr), arsenic (As) (Tiwari et al. 2008), iron (Fe), manganese (Mn), and zinc (Zn) (Eid & Shaltout 2016). Notably, *Portulaca oleracea* L. exhibits hyperaccumulation potential (> 1000 mg/kg) for several metals, such as Cu, Ni, Hg, Pb (Dwivedi et al. 2012), and Mn (Eid & Shaltout 2016). Pot experiments reinforce these capabilities. For instance, *Portulaca oleracea* L. demonstrated Cd removal efficiency ranging from 0.25 to 4.6%, with the most of the Cd accumulating in the aerial parts of the plant (Yousefi et al. 2023). Similarly, *Portulaca grandiflora* has shown the ability to accumulate metals like Al, Cd, Cr, Cu, Fe, Ni, Pb, and Zn, with hyperaccumulation properties for Al, Cu, Fe, and Zn (Vijayaraghavan et al. 2017). It also shown high tolerance to Cd contamination, accumulating significant amounts of Cd in its roots (62.4 μg/g), stems (150.5 μg/g), and leaves (90.5 μg/g), with a translocation factor of 5.45 and bioaccumulation factors (BAF) of 0.86 (Mahajan et al. 2024). These high TF and BAF values suggest that Portulaca is not only efficient at taking up cadmium but also capable of translocating it within the plant, reinforcing its potential as an effective phytoremediation agent for contaminated soils (Alyazouri et al. 2020; Dwivedi et al. 2012; Reboredo et al. 2019; Rehman et al. 2017).

*Portulaca oleracea*’s biomass production remains unaffected or even stimulated by heavy metal stress. In a pot experiment by Thalassinos et al. (2023), plants exposed to up to 900 mg Pb/kg accumulated up to 1,326 mg/kg Pb in roots and 133.5 mg/kg in shoots. Interestingly, plant growth improved with increasing Pb levels and nitrogen (N) addition, with shoot biomass increasing from 1.04 g (control) to 3.81 g under Pb and N co-application. This finding suggests a practical strategy of nutrient supplementation (e.g., N fertilization) to enhance metal uptake and biomass yield, thereby improving the overall phytoextraction efficiency (Thalassinos et al. 2023).

To enable practical field-level integration of *Portulaca* species in phytoremediation, they can be strategically cultivated in marginal or contaminated lands, particularly those unsuitable for food crops. Besides, intercropping or border planting with *Portulaca* in contaminated agricultural zones can localize and mitigate pollutant spread while maintaining some degree of agricultural productivity. Their ground-covering growth also contributes to erosion control and soil stabilization.

These results highlight the promising role of *Portulaca* species, particularly *Portulaca oleracea* L. and *Portulaca grandiflora*, in addressing heavy metal pollution in soils. Given their remarkable capacity to accumulate and translocate metals, these plants could be valuable tools in phytoremediation strategies aimed at cleaning up contaminated environments. However, further studies should focus on optimizing growth conditions and identifying the limits of metal uptake and accumulation, as well as assessing the long-term sustainability of using these species in large-scale remediation efforts. Additionally, exploring the genetic mechanisms behind their tolerance and hyperaccumulation properties could open new avenues for improving their efficiency and application in environmental management.

### Other uses

*Portulaca* species have demonstrated diverse uses besides their ecological and medicinal properties. In animal agriculture, *Portulaca* has shown promise as a valuable component of animal feed, contributing to improved livestock development in broiler chickens (Sadeghi et al. 2016; Wang et al. 2021) and fish (Şahin et al. 2022; Dahran et al. 2025). For instance, *Portulaca oleracea* L. supplementation at 2% and 3% in the diet of broilers significantly improved growth performance, with higher average daily weight gains of 1.44 g and 2.17 g, respectively (Wang et al. 2021). The 3% inclusion also reduced the feed conversion ratio by 0.26%, indicating improved feed efficiency. Additionally, *Portulaca oleracea* L. supplementation positively influenced the gut microbiota of broilers, notably by increasing the abundance of *Lactobacillus*, especially for Nile tilapia fish grown under a Cd exposure at 50 µg/L. This promotes gut health and nutrient absorption, which ultimately leads to improved weight gain (Sadeghi et al. 2016; Wang et al. 2021).

Similar benefits have been observed in aquaculture. For example, the addition of 9 g/kg *Portulaca* extracts to the diet of *Labidochromis caeruleus* resulted in a 0.51 g increase in weight gain, a 1.23% per day improvement in specific growth rate, and a survival rate of 83.33% (Şahin et al. 2022). Furthermore, *Portulaca* leaf powder (PLP) has been shown to mitigate the effects of heavy metal exposure in fish. In *Oreochromis niloticus*, 1% PLP supplementation led to a 46.11% greater weight gain than the unfortified controls. Under Cd exposure, PLP increased survival by 6.67%, and improved weight gain by 57.45% compared to non-supplemented fish. This protective effect was attributed to PLP’s ability to enhance antioxidant defense mechanisms by elevating intestinal catalase, SOD, and reduced-glutathione levels while reducing MDA content, thereby mitigating Cd-induced oxidative damage (Dahran et al. 2025). These findings highlight the potential of *Portulaca* spp. as a sustainable and functional feed additive, enhancing animal growth and feed efficiency.

*Portulaca* species are also valued in the gardening and landscaping sectors. *Portulaca grandiflora*, *Portulaca oleracea* L., *Portulaca umbraticola,* and *Portulaca pilosa* L. are a notable *Portulaca* species that is widely used for this purpose. These plants are valued for their vibrant, colorful flowers, and hardiness, requiring minimal maintenance compared to other ornamental plants. *Portulaca grandiflora* is highly favored for its rose-like flowers that bloom in various colors, earning it the common name “Japanese Rose”. Beyond its aesthetic appeal, *Portulaca oleracea* L. holds potential as a microgreen crop because of its exceptional nutritional profile. It is rich in minerals, antioxidants, ascorbic acid, phenolic compounds, and flavonoids, and offers numerous health benefits (Uddin et al. 2014). Consuming *Portulaca oleracea* L. has been linked to cardio-protection, anticancer effects, anti-inflammatory properties, and enhanced immune system support (Tungmunnithum et al. 2018; Corrado et al. 2021). This dual-purpose nature makes *Portulaca* a valuable plant for both ornamental and nutritional applications.

**Fig. 4:**
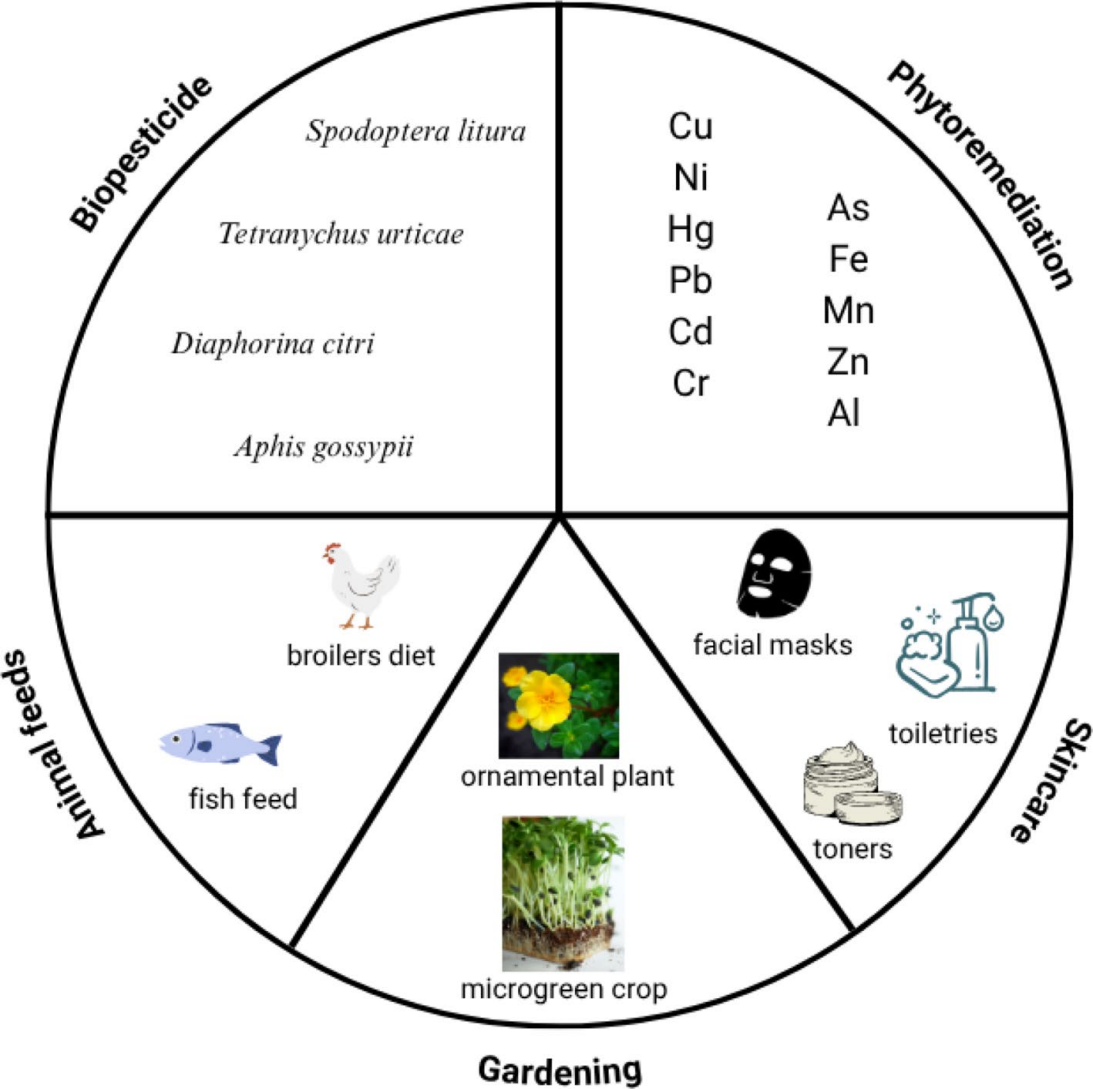
Overview of the multifaceted agricultural and commercial applications of *Portulaca* species. This diagram illustrates the diverse uses of *Portulaca*, particularly *P. oleracea*, across five domains: biopesticide (targeting pests like *Spodoptera litura* and *Tetranychus urticae*), phytoremediation (accumulating heavy metals, such as Pb, Cd, and Hg), animal feed (for broilers and fish), gardening (as ornamentals and microgreens), and skincare (in facial masks, toners, and toiletries). These applications highlight *Portulaca*’s versatility in sustainable agriculture, environmental remediation, and commercial industries

Furthermore, *Portulaca* extracts have gained substantial economic significance because of their versatile applications in multiple sectors, including facial masks, toners, and toiletries. By 2023, the global market value of Portulaca extracts was estimated to be USD 7,013 million, with North America, Europe, and Asia serving as the primary market regions (Valuates Reports 2024). This growing demand highlights the increasing value of plants in the cosmetic and personal care industries. Given its diverse benefits, *Portulaca* holds great promise as a multifunctional plant, but its full potential will only be realized through continued research and innovation across these varied fields. An overview of the agricultural applications of *Portulaca* is presented in Fig. 4.

## Abiotic stress tolerance in *Portulaca*

**Fig. 5:**
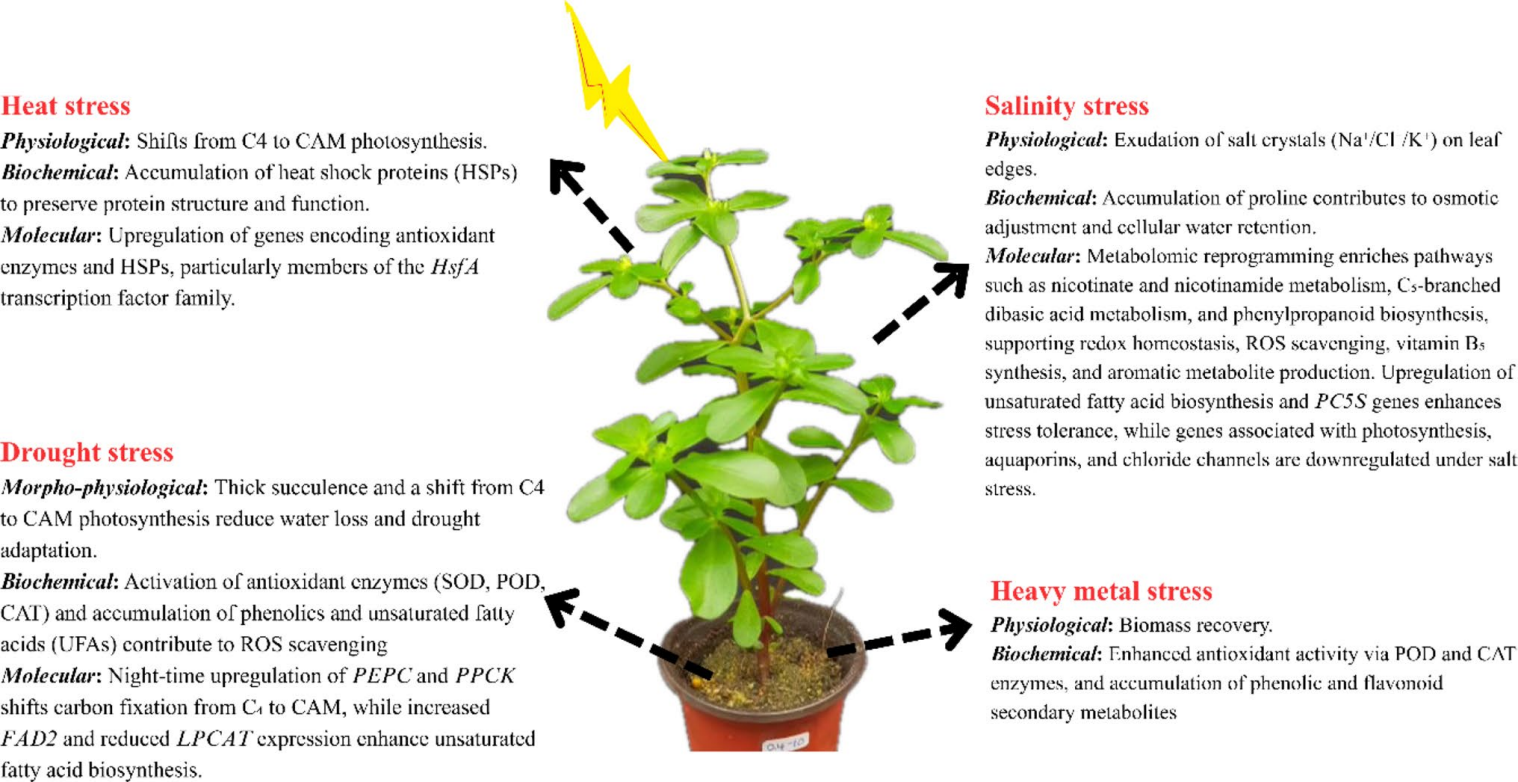
Graphical summary illustrates adaptation of *Portulaca* to various abiotic stressors. CAT, Catalase; FAD2, Fatty acid desaturase 2; HSPs, Heat shock protein; LPCAT, lysophosphatidylcholine acyltransferase; PEPC, phosphoenolpyruvate carboxylase; POD, Peroxidase; PPCK, Phosphoenolpyruvate carboxykinase; ROS, Reactive oxygen species; SOD, Superoxide dismutase; UFAs, Unsaturated fatty acids

Abiotic stresses, including drought, heat, and salinity, pose significant challenges to agricultural productivity by affecting plant growth, metabolism, and yield (Lau et al. 2022). Remarkably, *Portulaca* species demonstrate exceptional adaptability and resilience under such conditions, employing unique physiological, biochemical, and molecular mechanisms to survive and thrive (Fig. 5). This section explores the tolerance of *Portulaca* to abiotic stresses, emphasizing its ability to mitigate adverse environmental effects.

### Drought stress

The thick and fleshy leaves of *Portulaca* function as efficient water storage organs, providing a vital reserve that enables the plant to survive prolonged periods of drought or reduced water availability (Li et al. 2024b). In addition, the leaf surface exhibits pubescence of up to 1 mm in length (Uddin et al. 2014), which forms an air-trapping layer that reduces water evaporation and acts as an insulating layer, helping to lower leaf temperature (Sandquist and Ehleringer 1998). A waxy cuticle deposited on the leaf surface (Uddin et al. 2014) further limits molecular diffusion, including water loss (Arya et al. 2021). Together, these physical adaptations enhance *Portulaca*’s capability to survive under limited water availability or drought conditions.

*Portulaca* is a unique succulent plant capable of utilizing both C_4_ and CAM photosynthesis, an adaptation that was previously thought to be incompatible (Sage 2002). This remarkable photosynthetic plasticity allows *Portulaca* to switch between C₄ and CAM pathways depending on environmental conditions, particularly water availability. CAM involves the temporal separation of carbon fixation. At night, the stomata open to absorb carbon dioxide, which is stored as a 4-carbon compound, malic acid, in the vacuoles. During daylight, stomata remain closed to minimize water loss, and the stored malic acid is decarboxylated to supply CO₂ for the Calvin cycle (Borland et al. 2009). This mechanism is particularly effective under drought and heat stress, as it significantly reduces daytime transpiration (Amin et al. 2019). CAM has been reported to use up to 80% less water than C3 metabolism while producing comparable amounts of biomass (Shah et al. 2022).

Under well-watered conditions, *Portulaca* primarily employs the C₄ pathway, which involves spatial separation of carbon fixation between mesophyll and bundle sheath cells (Hatch-Slack pathway) to maximize carbon assimilation (Ferrari et al. 2020). However, during periods of drought or heat stress, the plant activates CAM metabolism, where carbon fixation is temporally separated within individual mesophyll cells to conserve water. This shift is supported by transcriptomic and gene co-expression analyses. For example, *Portulaca oleracea* L. and *Portulaca amilis* exhibit low basal levels of CAM activity under non-stress conditions but rapidly upregulate CAM-associated genes during water deficit (Gilman et al. 2022). In *Portulaca oleracea*, drought stress induced significant increases in the expression of CAM-related genes, with increases of up to 165-, 123-, and 25-fold in *PPC-1E1c*, *ALMT-12E.1*, and *DIC-1.1*, respectively, compared to well-watered plants (Ferrari et al. 2020). Upon re-watering, the expression of C_4_-related genes, including *PPC-1E1a’*, *NADME-1E.1*, and *ASPAT-1E1*, returned to levels comparable to those of well-watered treatments (Ferrari et al. 2020).

These transcriptional changes are closely tied to shifts in central carbon metabolism. Under drought, *Portulaca oleracea* L. exhibits reduced net CO₂ fixation and decreased levels of leaf monosaccharides (glucose, fructose, and galactose), indicating a slowdown in primary metabolism (D’Andrea et al. 2014). A hallmark of the C₄–CAM switch is the absence of CO₂ assimilation during the day and its uptake during the night (D’Andrea et al. 2014). This shift is accompanied by the upregulation of phosphoenolpyruvate carboxykinase (PPCK) at night, facilitating CO₂ fixation during the night and increases in malate accumulation (Gilman et al. 2022).

**Fig. 6:**
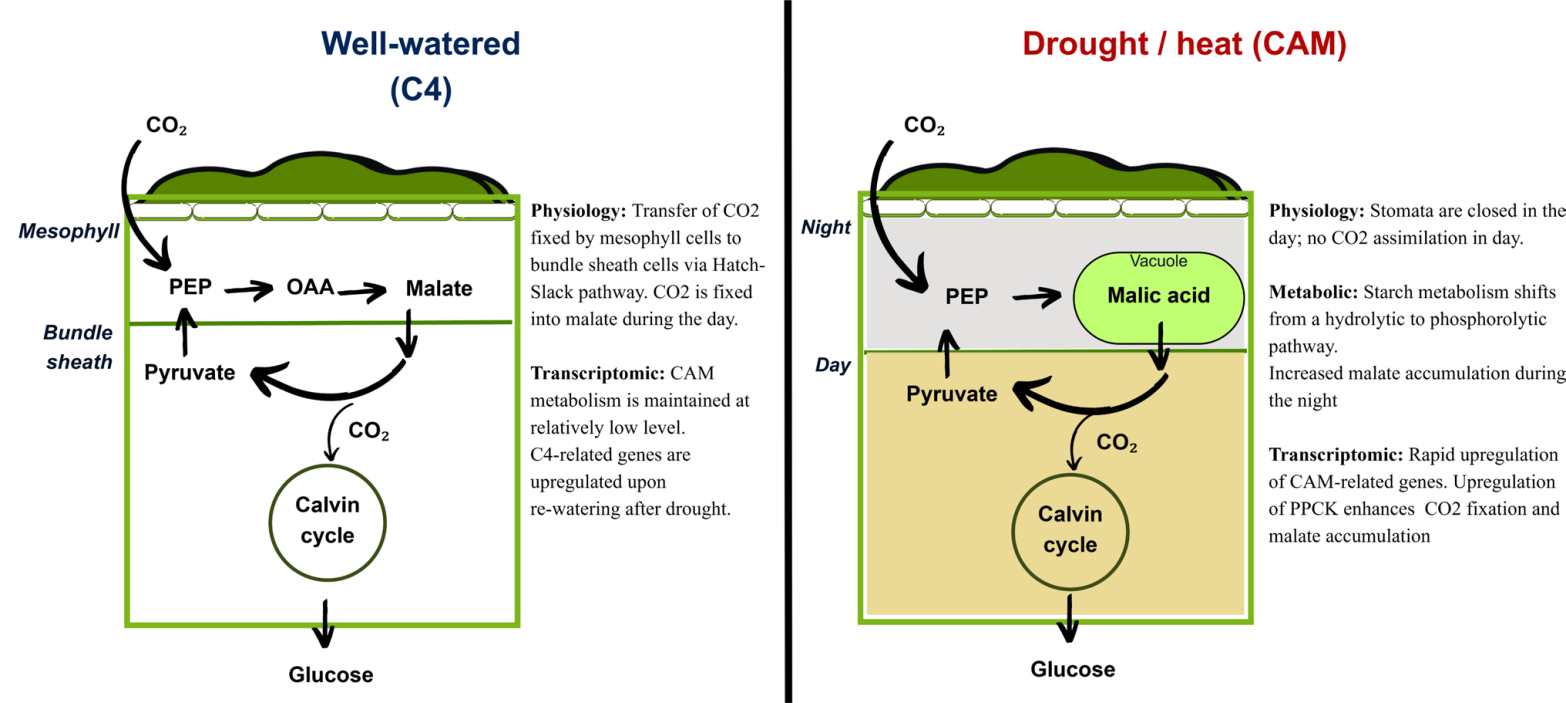
Graphical summary of C_4_-CAM plasticity in *Portulaca* under well-watered and drought- or heat-stressed conditions. OAA, Oxaloacetate; PEP, Phosphoenolpyruvate; PPCK, Phosphoenolpyruvate carboxykinase

The facultative CAM metabolic model predicts a transition in starch degradation pathways under drought. *Portulaca* shifts from hydrolytic (β-amylase) to a more energy-efficient phosphorolytic pathway mediated by α-glucan phosphorylase 2 under drought to conserve ATP for phosphoenolpyruvate regeneration (Gilman et al. 2022). A conserved CAM co-expression module associates a CAM-specific phosphoenolpyruvate carboxylase, PPC-1E1c’ ortholog, with starch degrading enzymes (glucan water dikinase and isoamylase) during the light-night transition, highlighting coordinated regulation between starch metabolism and CAM activation (Gilman et al. 2022). These findings underscore the resilience of *Portulaca*, highlighting its ability to switch between C_4_ and CAM photosynthesis to adapt to varying water availability and to mitigate the negative effects of drought stress. Figure 6 illustrates the C_4_–CAM changes of *Portulaca* under well-watered and drought- or heat-stressed conditions.

In addition to its plasticity in C_4_ and CAM photosynthesis, *Portulaca* also exhibits high tolerance to drought through enhanced ROS scavenging activities and solute accumulation. Drought stress typically leads to ROS production, which imposes oxidative stress on the plant cells (Mohd Amnan et al. 2021; Lau et al. 2021). Similar to other plants, *Portulaca* mitigates ROS accumulation by exhibiting elevated antioxidant activity, including superoxide dismutase, catalase, and peroxidase enzymes in plant cells. These antioxidants play a critical role in reducing oxidative damage under drought conditions (Lau et al. 2023). Importantly, *Portulaca* plants demonstrate strong resilience following drought stress, with the ability to restore their normal physiological bioactivity after re-watering (Jin et al. 2015; 2016).

Transcriptomic studies further reveal the molecular mechanisms underlying this stress response. D’Andrea et al. (2015) identified 202 drought-responsive differentially expressed genes (DEGs), representing about 7.2% of the *Portulaca oleracea* L. transcriptome. Among these, 107 DEGs were upregulated, and 51 were downregulated in drought-stressed plants compared to well-watered controls. Upon re-watering, 44 DEGs were identified. These genes are associated with translation, signal transduction, and protein modification. At the metabolic level, drought stress induces significant changes in lipid metabolism. Notably, the expression of Ω-6-fatty acid desaturase 2 (FAD2) is upregulated, enhancing unsaturated fatty acid synthesis, while lysophosphatidylcholine acyltransferase is downregulated, leading to increased level of polyunsaturated fatty acid (PUFA) (Mapelli-Brahm et al. 2020; Yin et al. 2024). These lipid remodeling processes help maintain membrane fluidity and photosynthetic efficiency under stress. Furthermore, *Portulaca* exhibits high production of phenolic compounds, which are known to protect cellular structures from oxidative damage (Tungmunnithum et al. 2018).

Together, these physiological, molecular, and metabolic adaptations have made *Portulaca* a vigorous plant to survive under water-deficient habitats and a model, especially in utilizing both C_4_ and CAM metabolism for boosting the development of drought-resilient agriculture crops.

### Heat stress

Heat stress significantly affects plant metabolism and disrupts photosynthesis, enzymatic activity, and cellular integrity. However, *Portulaca* has evolved several mechanisms to mitigate the adverse effects of elevated temperature. One of the key adaptations is cellular and molecular response, mainly through the production of heat shock proteins (HSPs). These molecular chaperones play a vital role in stabilizing membranes, preventing protein aggregation, and facilitating the proper folding of nascent polypeptides under thermal stress. HSPs also contribute to the detoxification of ROS by enhancing the activity of antioxidant enzymes (Amnan et al. 2022). Their expression is regulated at the transcriptional level through heat shock transcription factors, which are activated under high-temperature conditions (Ul Haq et al. 2019). Genomic analyses of *Portulaca oleracea* L. have identified five heat shock transcription factor (HsfA-family) paralogs, originating from both whole-genome duplication and tandem duplication events. All five genes were significantly upregulated under combined drought and heat stress, indicating their central role in promoting HSP biosynthesis and stress resilience (Wang et al. 2023).

As previously discussed, *Portulaca*’s ability to switch between C₄ and CAM photosynthesis significantly contributes to its heat tolerance. This metabolic flexibility allows the plant to optimize carbon assimilation and water-use efficiency under heat stress, thereby sustaining growth with minimal physiological disruption. For instance, *Portulaca* shows thermotolerance at 35 °C and 90% humidity, maintaining high photosynthetic activity with minimal oxidative damage (Yang et al. 2012). In the same study, Yang et al. (2012) identified 51 differentially expressed proteins under combined heat and humidity stress. These proteins were involved in various biological processes, including material and energy metabolism, antioxidant defense responses, protein destination and storage, and transcriptional regulation. Notably, antioxidant defense-related proteins are upregulated over time, reducing ROS accumulation and preventing cellular deterioration (Yang et al. 2012). Together, these physiological and molecular adaptations underscore the resilience and capacity of *Portulaca* spp*.* to thrive under heat stress.

### Salinity stress

*Portulaca* demonstrates resilience in high-salinity environments where many plants cannot adapt due to disrupted water uptake and toxic ion accumulation. For example, *Portulaca* maintained vigorous growth even under high-salinity conditions (15.2 dS/m), dominated by chloride and sulfate ions, while still producing high yields. It also demonstrated strong regrowth capability under irrigation with a selenium solution at 2.3 mg/L, further showing its adaptability to saline environments (Grieve and Suarez 1997). Another study demonstrated that *Portulaca* could sustain improved growth, including increased stem length, when exposed to sodium chloride concentrations of 50 and 100 mM NaCl. However, at 150 mM NaCl, both stem length and leaf number were reduced, indicating a threshold beyond which growth is negatively impacted (Sdouga et al. 2019). This resilience may be partly attributed to *Portulaca*’s salt exclusion mechanism, evidenced by the formation of visible white salt crystals (mainly identified to be composed of Na^+^, Cl^−^, and K^+^) on the leaf edges of salt-stressed *Portulaca oleracea* L. (Silva et al. 2023). Supporting this, metabolomic profiling of salt-stressed *Portulaca* identified 20 differentially expressed metabolites (DEMs) enriched in pathways, such as nicotinate and nicotinamide metabolism, C5-branched dibasic acid metabolism, and phenylpropanoid biosynthesis (Silva et al. 2023). The nicotinate and nicotinamide metabolism pathway plays a key role in regulating NAD⁺ and NADP⁺ homeostasis, which is essential for maintaining redox balance and other metabolic processes under salinity stress (Tamanna et al. 2024). The C5-branched dibasic acid metabolism, occurring in the chloroplast stroma, catalyzes the breakdown of branched-chain amino acids, such as valine, leucine, and isoleucine. Under salinity, branched-chain aminotransferase 2 (*OsBCAT2*) is upregulated, promotes branched-chain amino acids (BCAA) degradation and thus ROS scavenging activity and vitamin B5 biosynthesis to enhance salt tolerance (Sun et al. 2024). Phenylpropanoid biosynthesis involves phenylalanine synthesized via the Shikimate pathway, producing aromatic metabolites, including lignin, flavonoids, coumarins, phenolics, or tannins (Yu et al. 2025). These compounds play critical roles in plant development and stress responses (Geng et al. 2020).

At the gene expression level, salt stress triggers the down-regulation of several photosynthesis-related genes, such as chlorophyll a-b binding protein 6 (*CabBP6*), *CabBP4*, Photosystem II subunit P-1 (*PsbP1*), plastocyanin (*DRT112*), protochlorophyllide reductase (*PORA*), and ribulose bisphosphate carboxylase small chain (*RBCS*) (Xing et al. 2020). In addition, *Portulaca* shows significant down-regulation of aquaporins (*TIP1-1*, *TIP2-1*, and *PIP2-7*), which prevent excessive water loss by reducing water permeability, thereby maintaining cellular osmotic balance under salinity stress (Boursiac et al. 2005). Chloride channel proteins (CLA-a and CLA-b) are also downregulated in salt-stressed *Portulaca*, suggesting a mechanism to limit chloride ion uptake in chloride-rich environments and avoid chloride toxicity (Xing et al. 2020).

The resilience of *Portulaca* is further supported by its capacity for osmotic adjustment via proline accumulation. Proline acts as an osmoprotectant, and its levels increase proportionally with salinity exposure (Borsai et al. 2020). This response is supported by the upregulation of the *pyrroline-5-carboxylate synthetase (PC5S*) gene, which plays a crucial role in proline biosynthesis and is strongly associated with enhanced salinity tolerance (El Moukhtari et al. 2020). In addition to proline accumulation, *Portulaca* also exhibits significantly lower levels of malondialdehyde (MDA) compared to non-saline controls (Borsai et al. 2020). Since MDA is a widely recognized indicator of membrane damage that typically increases under salt stress (Hnilickova et al. 2021), the low MDA levels in *Portulaca* suggest its robust antioxidant defense and ability to sustain growth and development even in saline environments.

Salt tolerant *Portulaca* genotypes exhibit an upregulation of unsaturated fatty acid (UFA) biosynthesis-related genes (Du et al. 2023). Increased UFA content maintains plasma membrane fluidity and facilitates the proper function of Na⁺/H⁺ antiporters, which help maintain low cytosolic Na⁺ concentrations critical for salt tolerance (Allakhverdiev et al. 2001). Besides, 18-carbon UFAs can be further metabolized into signaling molecules, such as jasmonic acid, which orchestrates broad stress response pathways in plants (Wang et al. 2020b). Together, these findings indicate that *Portulaca* species possess strong adaptive defense mechanisms that enable them to tolerate high-salinity conditions.

### Heavy metal stress

*Portulaca* also exhibits notable tolerance to heavy metal stress, a growing environmental concern due to increased contamination from mining, industrial discharge, and agricultural runoff. Among various heavy metals, copper (Cu) is a common pollutant widely studied for its toxicity in plants (Izydorczyk et al. 2021). In a comparative study, seven out of nine *Portulaca* accessions maintained germination rates similar to controls even under exposure to 800 ppm of CuSO₄, demonstrating a high degree of Cu stress tolerance (Ren & White 2019). Three accessions of *Portulaca* showed no significant reduction in hypocotyl length at 100 ppm Cu, while five accessions maintained shoot and root growth at 600 ppm Cu. The Eritrea, P.O., and Turkey accessions performed particularly well under Cu stress, showing robust growth and low Cu accumulation in shoots under 600 ppm Cu (Ren & White 2019).

In another study, *Portulaca oleracea* L. exposed to heavy metal stress showed elevated oxidative stress markers, such as MDA and hydrogen peroxide. However, this was counteracted by enhanced antioxidant defenses mechanism, including the upregulation of peroxidase, catalase (CAT), as well as increased levels of phenolic compounds and flavonoids (Rahbarian et al. 2019). These responses suggest an active and effective oxidative stress mitigation strategy. Similarly, while Zn exposure led to some reduction in biomass in *Portulaca grandiflora*, the plant tolerated up to 2 mmol/kg of Ni, Pb, and Zn both individually and in paired combinations (Ni + Pb, Ni + Zn, and Pb + Zn). This tolerance was associated with significantly elevated proline accumulation and a modest increase in lipid peroxidation (MDA) (Mihailovic et al. 2015). Collectively, these findings highlight *Portulaca*’s robust defense strategies under heavy metal stress and underscore its potential utility in phytoremediation efforts.

## Conclusion and future prospect

*Portulaca* species represent a versatile and resource-efficient plant genus with significant potential across agriculture, medicine, and environmental sustainability. Their rich phytochemical profile, adaptability to harsh environments, and multifunctional applications highlight their importance in addressing global challenges, such as food security, healthcare, and climate resilience. *Portulaca oleracea* L., in particular, has been well documented for its nutritional and pharmacological value. However, a broader understanding of the genus remains limited, as most research has focused on a single species. Unlocking the full potential of *Portulaca* requires a more systematic exploration of its lesser-known species, integrative approaches to improve metabolite yields, and strategic implementation of its ecological and commercial applications.

Despite significant advancements in the study of *Portulaca*, several key areas remain underexplored. Most existing studies have focused predominantly on *Portulaca oleracea* L., leaving the phytochemical diversity and adaptive traits of other species in the genus largely unknown. Expanding research efforts to include these lesser-known species may lead to the discovery of novel bioactive compounds and unique stress tolerance mechanisms that could be harnessed for agricultural or pharmaceutical purposes. Advances in multi-omics technologies, including genomics, transcriptomics, proteomics, and metabolomics, now offer powerful tools to unravel the biosynthetic potential of *Portulaca* species. Combined with synthetic biology approaches, such as CRISPR-Cas9 genome editing and metabolic engineering, these technologies can be used to identify and enhance the production of high-value secondary metabolites. This integrative approach will not only improve metabolite yields but also pave the way for more cost-effective and scalable applications in industry and healthcare.

Numerous studies have reported the antioxidant, anti-inflammatory, antimicrobial, and anticancer properties of compounds derived from *Portulaca*. However, only few have undergone rigorous toxicological evaluation or clinical validation. To ensure the safe and effective therapeutic use of *Portulaca*-based products, future research must prioritize comprehensive toxicological assessments, preclinical investigations, and well-designed clinical trials. These efforts will be critical for substantiating health claims, gaining regulatory approval, and fostering consumer confidence.

Optimizing cultivation practices is another pivotal focus area. Refining growth conditions, such as soil amendments, irrigation strategies, and nutrient management, could significantly increase biomass yields and enhance phytochemical content. Furthermore, integrating *Portulaca* into agroecological systems, such as intercropping or agroforestry, may increase ecosystem resilience and contribute to sustainable farming practices. Additionally, investigating how environmental stressors influence the biosynthesis of bioactive compounds could unlock additional therapeutic and economic value, reinforcing the role of *Portulaca* in sustainable food systems and natural health solutions.

Equally important is the development of scalable phytoremediation models is crucial for fully realizing *Portulaca*’s environmental benefits. Long-term studies evaluating the viability and safety of using *Portulaca* in heavy metal-contaminated sites are essential for ensuring its effectiveness. Advancing *Portulaca*’s role in phytoremediation requires robust collaboration across multiple sectors. By fostering partnerships between academic researchers, industry stakeholders, and regulatory agencies, we can expedite the translation of laboratory discoveries into practical, large-scale solutions. For example, industry partners can contribute by designing cost-effective, innovative technologies, while regulatory agencies are critical in ensuring safety and compliance with environmental standards.

## Data Availability

No datasets were generated or analyzed during the current study.
